# Profiling of 3D Genome Organization in Nasopharyngeal Cancer Needle Biopsy Patient Samples by a Modified Hi-C Approach

**DOI:** 10.3389/fgene.2021.673530

**Published:** 2021-09-03

**Authors:** Sambhavi Animesh, Ruchi Choudhary, Bertrand Jern Han Wong, Charlotte Tze Jia Koh, Xin Yi Ng, Joshua Kai Xun Tay, Wan-Qin Chong, Han Jian, Leilei Chen, Boon Cher Goh, Melissa Jane Fullwood

**Affiliations:** ^1^Cancer Science Institute of Singapore, Centre for Translational Medicine, National University of Singapore, Singapore, Singapore; ^2^School of Biological Sciences, Nanyang Technological University, Singapore, Singapore; ^3^Department of Haematology-Oncology, National University Cancer Institute, National University Health System, Singapore, Singapore; ^4^Department of Otolaryngology - Head and Neck Surgery, National University of Singapore, Singapore, Singapore; ^5^Department of Anatomy, Yong Loo Lin School of Medicine, National University of Singapore, Singapore, Singapore; ^6^Department of Pharmacology, Yong Loo Lin School of Medicine, National University Health System, Singapore, Singapore

**Keywords:** nasopharyngeal cancer, chromatin organization, Hi-C, Biop-C, 3D genome organization

## Abstract

Nasopharyngeal cancer (NPC), a cancer derived from epithelial cells in the nasopharynx, is a cancer common in China, Southeast Asia, and Africa. The three-dimensional (3D) genome organization of nasopharyngeal cancer is poorly understood. A major challenge in understanding the 3D genome organization of cancer samples is the lack of a method for the characterization of chromatin interactions in solid cancer needle biopsy samples. Here, we developed Biop-C, a modified *in situ* Hi-C method using solid cancer needle biopsy samples. We applied Biop-C to characterize three nasopharyngeal cancer solid cancer needle biopsy patient samples. We identified topologically associated domains (TADs), chromatin interaction loops, and frequently interacting regions (FIREs) at key oncogenes in nasopharyngeal cancer from the Biop-C heatmaps. We observed that the genomic features are shared at some important oncogenes, but the patients also display extensive heterogeneity at certain genomic loci. On analyzing the super enhancer landscape in nasopharyngeal cancer cell lines, we found that the super enhancers are associated with FIREs and can be linked to distal genes *via* chromatin loops in NPC. Taken together, our results demonstrate the utility of our Biop-C method in investigating 3D genome organization in solid cancers.

## Introduction

The three-dimensional (3D) genome organization of the nucleus plays a vital role in the regulation of transcription (Hnisz et al., 2016, 2018). Alterations in 3D genome organization structures including topologically associated domain boundaries and chromatin loops have been shown to lead to oncogene expression and cancer progression (Fudenberg et al., 2011; Flavahan et al., 2016; Hnisz et al., 2016; Valton and Dekker, 2016; Li et al., 2019).

High-throughput chromosome conformation capture technologies such as Hi-C have been used to investigate 3D chromatin conformation (Lieberman-Aiden et al., 2009; van Berkum et al., 2010). The standard Hi-C approach generally requires approximately one million cells. Consequently, most previous analyses of cancer samples have been restricted to human cancer cell lines (Rao et al., 2014; Darrow et al., 2016; Haarhuis et al., 2017), but recently, Hi-C has been conducted on clinical samples from liquid cancers such as T-cell acute lymphoblastic leukemia (T-ALL) (Kloetgen et al., 2020) and diffuse large B-cell lymphoma (DLBCL) (Díaz et al., 2018) and one solid cancer—gastric cancer (Ooi et al., 2020). For these cancers, it is possible to obtain one million cells—for example, gastric cancers can grow to a large size. However, there are many cancers for which only needle biopsies are available (Lin et al., 2017).

To allow interrogation of samples with more limited quantities of starting materials, there have been several efforts to reduce the number of cells required to just 1K or 500 cells using modified protocols such as small-scale *in situ* Hi-C (sisHi-C) (Du et al., 2017), easy Hi-C (Lu et al., 2020), and Low-C (Díaz et al., 2018) (Supplementary Table 1). However, when dealing with solid cancers, a second challenge is that the tissue requires special preparation in order to dissociate the tissue into single cells for Hi-C analysis. The core needle biopsies pose the challenge of both limited cell numbers as well as the requirement for tissue dissociation, which might lead to further loss or degradation of chromatin for analysis. Solid cancers represent approximately 90% of adult human cancers (American Cancer Society, 2020); therefore, an easy-to-use method for preparing Hi-C libraries from needle biopsy cancer samples would advance our understanding of how chromatin organization contributes to cancer pathogenesis in solid cancers.

Here, we present Biop-C, a modified *in situ* Hi-C method for the chromatin analysis in solid cancer tissues from needle biopsy samples. The Biop-C method has been designed to be used on small tissue samples obtained from needle biopsies. To demonstrate the utility of this method, we analyzed three nasopharyngeal cancer (NPC) patient samples. NPC is an epithelial malignancy of the nasopharyngeal mucosa and is an aggressive subtype of head and neck cancers. NPC is highly prevalent in Southeast China, Southeast Asia, North Africa, Middle East, and the Arctic regions, but rare in most other parts of the world (Yu and Yuan, 2002). Multiple factors, including predisposing genetic factors, environmental carcinogens, and Epstein–Barr virus (EBV) infection, contribute to the etiology of NPC (Li et al., 2014; Dai et al., 2016). NPCs are further subdivided into three subtypes, viz. non-keratinizing undifferentiated carcinoma, non-keratinizing differentiated carcinoma, and keratinizing squamous cell carcinoma. Depending on the treatment given, the stage of the cancer, and the site where the cancer presents at, NPCs can be small and analyzed using fine needle biopsies.

It has been established that NPC has a comparatively low mutational burden, and oncogenicity is driven by epigenetic regulation. Typically, NPCs associated with EBV are characterized as having comparatively low DNA mutation rates but widespread DNA hypermethylation and overexpression or mutation of DNA methylation enzymes, histone modification enzymes, and chromatin remodeling enzymes (Dai et al., 2015, 2016; Tsang et al., 2020). Hence, unraveling the 3D conformational structure will provide further insight into the epigenetic regulatory mechanisms that promulgate the NPC phenotype. Here, we obtained a comprehensive understanding of the 3D genome organization of nasopharyngeal cancer through Biop-C analysis, which revealed complex 3D genome organization patterns at oncogenes important in NPC.

Moreover, as cancers are known to be heterogeneous but chromatin interaction heterogeneity in patient samples is poorly understood, we investigated chromatin interactions in three different nasopharyngeal cancer samples. We found that while there were similar chromatin interactions in all three samples, there were also chromatin interactions that were heterogeneous. We also prepared Hi-C libraries from an NPC cell line, HK1, and found differences between chromatin interactions in the patient samples as compared with the cell line, indicating the necessity of interrogating chromatin interactions in actual patient cancers, which Biop-C enables. Taken together, our results demonstrate the utility and importance of Biop-C as a method for understanding cancer 3D genome organization.

Additionally, we analyzed how NPC chromatin interactions differ from that of a near-normal nasopharyngeal cell line, NP-69, and we analyzed how THZ1, a drug that inhibits super enhancers, leads to changes in chromatin interactions in the NPC cell line, HK1. These functional data suggest that there are differences in the chromatin interaction landscape between nasopharyngeal cancer and normal nasopharyngeal cell line, and targeting super enhancers by THZ1 can modulate the chromatin interactome and lead to losses in chromatin interactions, suggesting that epigenetic drugs may be able to affect chromatin interactions that are altered in nasopharyngeal cancer.

## Materials and Methods

### Cell Culture

Nasopharyngeal cancer HK1 cells were cultured in Roswell Park Memorial Institute (RPMI) 1640 media (Hyclone) supplemented with 10% heat-inactivated fetal bovine serum (FBS; Hyclone) and 1% penicillin/streptomycin (Hyclone). NP-69 cells were cultured in keratinocyte SFM media. All cell lines were grown in an incubator at 5% CO_2_ and 37°C. The HK1 and NP-69 cell lines were a kind gift from Prof. Goh Boon Cher, Cancer Science Institute, National University of Singapore.

### Biopsy Samples

Nasopharyngeal cancer patient samples were obtained from the National University Health System (NUHS) with patient consent, under Institute Review Board number 2018/00947-SRF0002. The clinical samples were collected by needle biopsy in a 1.5-μl microtube by trained clinicians. The needle type varies depending on the size and the location of the tumor. All clinical samples were obtained from the National University Hospital Singapore and collected according to the Human Biomedical Research Act requirements. Informed consent was obtained for all clinical samples used in the study. The clinical details of the patients are listed in Supplementary Table 2.

The needle biopsy samples were flash frozen in liquid nitrogen immediately and stored at −80°C until further use.

### Pulverization of the Needle Biopsy Sample

The liquid nitrogen-cooled mini mortar and pestle (SP Scienceware, United States) were used for the pulverization of the fine needle biopsy sample of nasopharyngeal patients. The stainless-steel mortar and pestle were cooled on dry ice before use. The liquid nitrogen was poured into the steel cavity up to the mark indicated.

The samples were taken out of the freezer and immediately placed in the fixture of the mortar. The sample was kept frozen throughout the pulverization. A small amount of liquid nitrogen (up to half of the microtube) was carefully poured into the microtube. Then, the liquid nitrogen was allowed to evaporate just enough for the small biopsy sample to stay submerged. The sample was pulverized in the mortar using the precooled pestle. The above steps were repeated until the sample resembled a fine powder without visible chunks. Generally, it took three reiterations to pulverize a needle biopsy sample to a fine powder.

Finally, a cooled spatula was used to transfer any remaining pulverized tissue from the pestle into the 1.5-μl microtube. The microtube was submerged in dry ice to keep all the pulverized tissue frozen.

### Biop-C Library Preparation and Sequencing

Next, the Biop-C library was generated using the Arima Hi-C kit, according to the protocols of the manufacturer with slight modifications.

Specifically, the microtube was then removed from the dry ice, and the powder was mixed with 500 μl of 1× PBS. The chromatin was cross-linked by adding 50 μl of freshly prepared TC buffer (Arima kit; sodium chloride: 100 mM, EDTA: 1 mM, EGTA: 0.5 mM, HEPES pH 8.0: 50 mM, formaldehyde: 2%, water).

The samples were then mixed well by inverting and incubated at room temperature for 20 min. Finally, the reaction was stopped by adding stop solution (Arima kit). The pellet obtained after centrifugation was stored at −80°C until further use.

The fixed cells were permeabilized using a lysis buffer supplied in Arima Hi-C kit and then digested with a restriction enzyme cocktail. The resulting overhangs were filled in with biotinylated nucleotides followed by ligation. After ligation, crosslinks were reversed, and the DNA was purified from protein. Purified DNA was treated to remove biotin that was not internal to ligated fragments. The purified proximity-ligated DNA was sheared using a Covaris ME220 ultrasonicator.

The DNA fragments were size selected from ∼200 to 600 bp using DNA purification beads (AMPure XP beads). The size-selected fragments were then enriched for biotin and converted into Illumina-compatible sequencing libraries using low input Swift Biosciences Accel-NGS 2S Plus DNA Library Kit (Cat # 21024) and Swift Biosciences Indexing Kit (Cat # 26148). After adapter ligation, DNA was PCR amplified and purified using AMPure XP beads. The purified DNA underwent standard QC according to Arima Hi-C kit instructions such as qPCR and Agilent Bioanalyzer. Finally, the libraries were sequenced 150 bases paired-end on the Illumina HiSeq 4000 following the protocols of the manufacturer.

### Analysis of HiC Sequencing Data Using Juicer and FitHiC2 Pipelines

Hi-C data were processed using Juicer (version 1.5.7) (Rao et al., 2014) with default parameters. The reference genome used for mapping the reads was hg19. Reads with mapping quality under 30 were discarded. The quality data and statistics for Hi-C analysis can be found in Supplementary Table 3.

TADs were called using the Juicer tool called Arrowhead with 10 kb resolution. The normalization used for TAD calling is Knight-Ruiz (KR). The list of TADs called for each patient sample and HK1 cell line can be found in Supplementary Table 4.

Chromatin loops were identified using the Juicer tool called HiCCUPS. Loops were called for three resolutions: 5, 10, and 25 kb. The normalization used is KR. The list of loops called for each sample and HK1 can be found in Supplementary Table 5.

FitHiC2 was additionally used for the estimation of contact significance (Kaul et al., 2020). Resultant hic files produced by Juicer were converted to FitHiC2-compatible sparse matrices. Biases were estimated from the matrix, excluding 20% of the lowest contact frequency bins. Sparse matrices and bias files were subsequently passed to FitHiC2.

### THZ1 Treatment

The CDK7 inhibitor THZ1 (A8882) was purchased from ApexBio. The HK1 cells were grown overnight and then treated with THZ1 at 200, 500, and 1,000 nM concentrations for 24 h. The RNA was isolated from all three time points for real-time quantitative PCR (RT-qPCR). The Hi-C was performed from the cells treated at 500 nM for 24 h.

### RNA Extraction, cDNA Synthesis, and Real-Time Quantitative PCR

Total RNA was extracted from the THZ1-treated and DMSO-treated HK1 cells using RNeasy Mini Kit (Qiagen) with on-column DNase digestion (Qiagen) according to the instructions of the manufacturer. The cDNA was synthesized using the qScript^TM^ cDNA Synthesis Kit (Quanta Biosciences, MA, United States). RT-qPCR was conducted with GoTaq DNA Polymerase Master Mix (Promega, United States).

### FIRE Calling

FIRE calling was carried out using the FIREcaller software (Crowley et al., 2021). The resolution for FIRE calling was 10 kb. Dense Hic matrix for each chromosome was created using Juicer dump with KR normalization and was then converted to mcool format using script from FIREcaller. Frequently interacting regions (FIREs) which were either overlapping or book-ended were merged together using bedtools merge.

### Structural Variants Calling

Structural variants were called using the HiNT software (Wang et al., 2020). HiNT pre was used for preprocessing of Hi-C data and normalization followed by HiNT TL which is used for calling translocations and breakpoint detection. HiNT TL outputs candidate translocated chromosomal pairs and the exact location of the breakpoint (Supplementary Table 8). In case of an unmappable region, HiNT TL provides a 100-kb interval for the breakpoint.

### AB Compartment Analysis

The AB compartments were identified using FAN-C 0.9.1 (Kruse et al., 2020) at 500 kb resolution. Fanc compartments function was used for calling the AB domains and eigenvector values for each domain. Genome file was provided using -g command for assignment of domains. The eigenvector is oriented in such a way that negative entries correspond to “B” (low GC content, inactive compartment) and positive entries to “A” (high GC content, active compartment).

For analyzing the changes between A/B compartments of two samples, we calculated Pearson correlation coefficient.

### Super Enhancer and Enhancer Calling

ChIP-Seq data were aligned to reference genome hg19 using bwa mem (Li, 2013) with default parameters. PCR duplicated and blacklisted regions that fall under ENCODE consensus were removed using samtools markdup and bedtools intersect. Narrow peaks were then called using MACS2 (version 2.1.2) (Zhang et al., 2008). The modified version of ROSE package (Lunardon et al., 2014; Cao et al., 2017) was used for calling super enhancer stitched at 12 kb stitching distance. For calling individual enhancers, we overlapped stitched peaks with H3k27ac narrow peaks and the intersection was called as an enhancer. Super enhancers (SE) and enhancers from all the three cell lines were merged together using bedtools merge with a cutoff of 1 bp overlap. SE and enhancers present in all the three cell lines were identified as “common.”

### Comparison of TADs and Loops Between the Patients

Jaccard index was calculated using bedtools Jaccard for all the three patient samples as well as HK1 samples followed by comparison using bedtools overlap to identify the exact number of TADs which are common in two samples. TAD overlap of 80% was used as cutoff for characterizing two TADs as the same. The compare lists analysis of Juicer pipeline was used to compare the chromatin loops between the samples.

### Associating SE and Enhancers With Chromatin Loops and FIREs

Loop anchors within 15 kb distance from a super enhancer or an enhancer were recognized as an associated chromatin loop and the gene within 15 kb distance from the other anchor of the same loop was called as an associated gene. Similarly, SE and enhancers were also associated with FIREs *via* chromatin loops. Bedtools closest was used to identify features within 15 kb distance from SE.

### Comparison of Replicates and Other Hi-C Data

Hi-C data from sample replicates as well as different samples were compared using hicrep (Yang et al., 2017) with a resolution of 100 kb. For the analysis, cool files were generated using hic2cool^1^ and was run with default settings. Hicrep outputs stratum-adjusted correlation coefficient (SCC) as a similarity measure of Hi-C interaction matrices. SCC shares a similar range and interpretation as the standard correlation coefficients and the value lies between 1 and (−1).

### Enhancer–Promoter Interaction Analysis

For a given set of loops, we identified paired features (enhancers on one anchor and promoters on the other), by searching for features within 15 kb of the loop anchor centroids. The transcription start site was used to approximate the promoter position.

### Gene Set Overrepresentation Analysis

For the analysis of the NPC samples, we considered the intersection of genes looping to super enhancers, with promoters within 15 kb of loop anchor centroids, that were identified in all three NPC samples (S009, S010, and S012). For the analysis of HK1 following the THZ1 treatment paradigm, we considered the set of genes with promoters within 15 kb of loop anchor centroids, that showed a net loss in the number of associated loops following THZ1 treatment. For a given set of genes, significantly overrepresented Biological Process Gene Ontology (GO) terms were identified using the PANTHER 16.0 GSOA webtool (Mi et al., 2021). Statistical significance was estimated using a binomial test with false discovery rate (FDR) correction. Resultant enriched GO terms were used for subsequent GO set and network analysis using the NaviGo webtool (Wei et al., 2017).

## Results and Discussion

### A Genome-Wide Map of 3D Genome Organization in Nasopharyngeal Cancer

We applied “Biop-C” to analyze three NPC tissue samples, i.e., “S009,” “S010,” and “S012.” The tumor cores were collected by needle biopsies and immediately flash-frozen in liquid nitrogen (Supplementary Figure 1). The tumor cores were approximately weighed in the range 3–10 mg, and the clinical characteristics of the samples are listed in Supplementary Table 2. To prepare the tissue for analysis of 3D genome organization, we used a liquid nitrogen-cooled mini mortar and pestle for the pulverization of tissue within a microtube. This approach kept the biopsy sample frozen and reduced the risk of sample degradation. Additionally, this approach also provided the flexibility of performing the workflow from sample acquisition to Hi-C library preparation in a single microtube, which further minimized potential sample losses.

**FIGURE 1:**
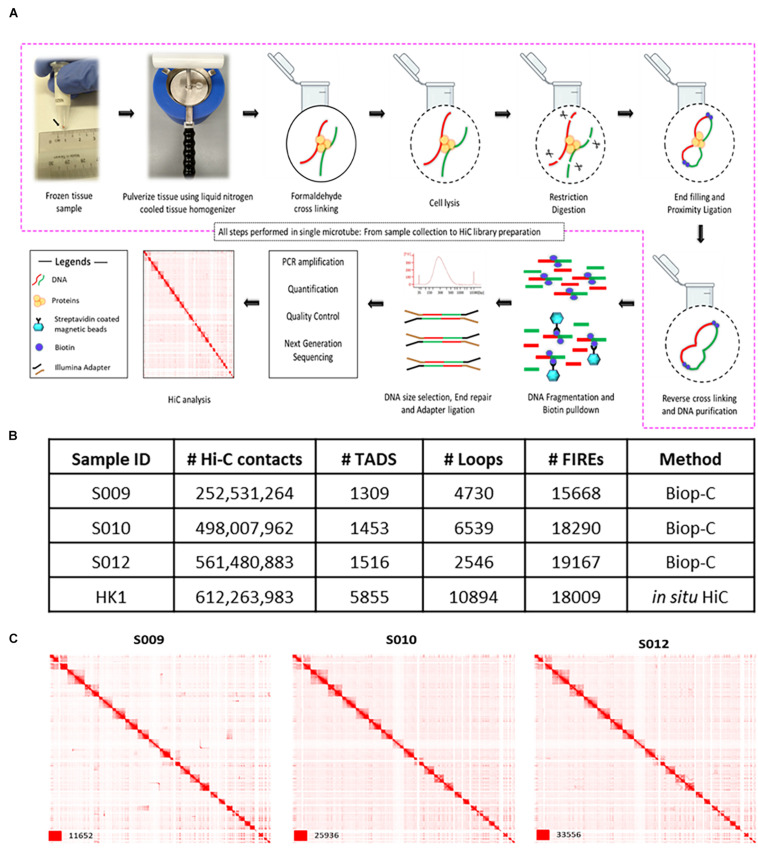
Biop-C method enables the study of high-resolution 3D genome organization and chromatin interactome in solid tumor samples. **(A)** Schematic overview of the Biop-C method. The frozen needle biopsy tissue is pulverized in a liquid nitrogen-cooled mini mortar, and then chromatin was fixed with formaldehyde in the nucleus. The samples were processed with commercial Arima genomics Hi-C kit. Fixed cells were permeabilized using a lysis buffer supplied in Arima Hi-C kit and then digested with a restriction enzyme. Following restriction digestion, the sites were filled in with biotinylated nucleotides. The resulting DNA blunt ends were subsequently ligated. After ligation, crosslinks were reversed to remove proteins from DNA. Hi-C material was then sonicated using a Covaris Focused-Ultrasonicator M220 instrument to achieve fragment sizes of 300–500 bp. The sonicated DNA was double-size selected using AMPure XP beads, and the sequencing libraries are generated using low input Swift Bioscience Illumina-compatible adapters. **(B)** Table of statistics of Biop-C and Hi-C data. #Hi-C contacts indicate the number of mapped/valid junction reads of each library. #TAD indicates the number of TADs called from Biop-C and Hi-C data at 10 kb resolution. #Loops indicates the total number of loops called from Biop-C and Hi-C data at 5, 10, and 25 kb resolution and then merged. #FIREs indicates the number of FIREs called from Biop-C and Hi-C data at 10 kb resolution. **(C)** Juicebox (Durand et al., 2016) visualized Biop-C and Hi-C heatmaps showing all chromosomes for S009, S010, S012, and HK1 cell line.

After pulverization, we processed the samples with a commercially available Arima *in situ* Hi-C kit. Briefly, chromatin was fixed with formaldehyde in the nucleus and digested with a restriction enzyme. Then, overhangs were filled in with biotinylated nucleotides followed by proximity ligation. After ligation, crosslinks were reversed, and the DNA was purified from protein. Furthermore, the purified DNA was sheared to ∼350 bp mean fragment size. Finally, the sequencing libraries were generated using low input swift bioscience Illumina-compatible adapters (Figure 1A). The usage of the mini mortar and pestle followed by Hi-C is the key innovation of the Biop-C method. While this improvement in sample processing is a small change, it is highly effective in generating high-quality chromatin interaction libraries from needle biopsy clinical samples (Figure 1C).

Finally, we sequenced Biop-C libraries deeply by Illumina next-generation sequencing using a HiSeq4000 machine. Each library contained between 450 and 922 M contacts (Supplementary Table 3). We obtained more than 200 million mapped/valid junction reads (>50% of total read pairs) for each library, reflecting that our Biop-C datasets are adequately complex (Lajoie et al., 2015) (Figure 1B and Supplementary Table 3). Furthermore, the low ratios of the number of *trans* to *cis* contacts indicate high library quality for all samples (Nagano et al., 2015) (Supplementary Table 3). Notably, in some samples (e.g., S009), only one lane of HiSeq4000 sequencing was sufficient to obtain a high-quality Biop-C library.

Additionally, for comparison with a typical Hi-C library, we generated two replicates of HK1 NPC cell line by traditional *in situ* Hi-C Arima kit (Dixon et al., 2012; Rao et al., 2014). Moreover, for comparison with normal nasopharynx, we generated two replicates of NP-69 near-normal nasopharynx cell line by traditional *in situ* Hi-C.

We used Juicer for processing the resulting data, and the package Arrowhead was used to annotate TADs genome-wide, while the package HiCCUPS was used to call loops (Rao et al., 2014). Heatmaps were visualized with Juicebox (Durand et al., 2016). We were able to successfully call TADs and loops from our libraries (Figure 1B and Supplementary Tables 3, 4), permitting comprehensive mapping of putative super and typical enhancer–promoter interactions in these samples (Supplementary Table 6). The TADs were clearly identifiable at a resolution of 10 kb. A high number of significant chromatin interactions could also be called using FitHiC2 (Kaul et al., 2020) at a resolution of 50 kb (Supplementary Table 7).

Patient S009 had 1,309 TADs, while patient S010 had 1,453 TADs and patient S012 had 1,516 TADs (Figure 1B). Loops could be identified at 5, 10, and 25 kb resolutions in all datasets, and these loops were all merged together for subsequent analyses. Patient S009 had 4,730 merged loops, while patient S010 had 6,539 merged loops, and patient S012 had 2,546 merged loops (Figure 1B).

We also called TADs and loops for NP-69 and found 5,227 TADs and 11,415 loops (Supplementary Figure 4). To better understand how NPC chromatin loops differ from normal loops found in a near-normal nasopharyngeal cell line, we compared chromatin loops in NP-69 with the ones called in NPC Biop-C samples. For comparison, we aggregated loops from the three samples (S009, S010, and S012) and compared them with NP-69 loops. We found that 4,026 loops (39.6% of aggregated NPC loops) were similar in NPC samples and NP-69. By contrast, 6,158 (60.4% of aggregated NPC loops) were specific to NPC, and 7,388 (72.5% of NP-69 loops) loops were specific to NP-69. We then associated these loops with gene promoters and found that the NP-69-specific loops (7,388) were associated with 11,167 gene promoters and the NPC-specific loops (6,158) were associated with 6,303 gene promoters (Supplementary Figure 4). We also found some examples of genes which were only associated with loops in NPC like *MMP3*, *CASP3*, and *ULK1* (Supplementary Figure 4). Overall, our results indicate that while some loops are similar between NP-69 near-normal cell line and NPC samples, there are also many loops that differ between them, suggesting that these may be cancer-specific loops that regulate oncogenes.

We looked for structural variants in biopsy samples S009, S010, and S012 as well as cell lines HK1 and NP-69. In S009, five translocations were identified out of which four can be seen in Hi-C heatmaps as well (Supplementary Table 8 and Supplementary Figure 5A). In S010, no translocation was recognized, and in S012, we found six translocation incidences and all of these can also be seen in Hi-C heatmap (Supplementary Table 8 and Supplementary Figures 5B,C). In cell lines, we found 63 translocation incidences in HK1 and 23 in NP-69 (Supplementary Table 8). Many of these translocations can be very clearly seen in the Hi-C heatmaps (Supplementary Figures 5D,E).

We could also recognize A/B compartments in our Biop-C samples (Supplementary Table 9 and Supplementary Figures 6A–C) as well as in NP-69 cell line (Supplementary Figure 6D). We then wanted to compare these A/B compartments within each sample as well as NP-69 by calculating the Pearson correlation coefficient (Pearson’s *r*) of the eigenvalues called with 500 kb resolution (Supplementary Table 9). The Pearson’s *r* for S009 compared with S010 is 0.83, for S009 and S012 0.74, and for S010 and S012 0.85. We then compared the eigenvalues between NP-69 and biopsy samples. The Pearson’s *r* for NP-69 compared with S009, S010, and S012 is 0.60, 0.64, and 0.54, respectively (Supplementary Figures 6E–J). These results show that the correlation between A/B compartments within biopsy samples is higher as compared with NP-69.

Moreover, because “FIREs” are a new type of chromatin interaction landmark associated with super enhancers and tissue-specific chromatin interactions (Schmitt et al., 2016), we used FIREcaller R package (Crowley et al., 2019) to call FIREs. We identified 2,783 FIREs in NPC sample S009, 1,393 FIREs in sample S010, and 2,906 FIREs in sample S012 from our Biop-C data. We also called FIREs from the NPC cell line HK1 and identified 3,585 FIREs (Supplementary Table 10 and Figure 1B).

**FIGURE 2:**
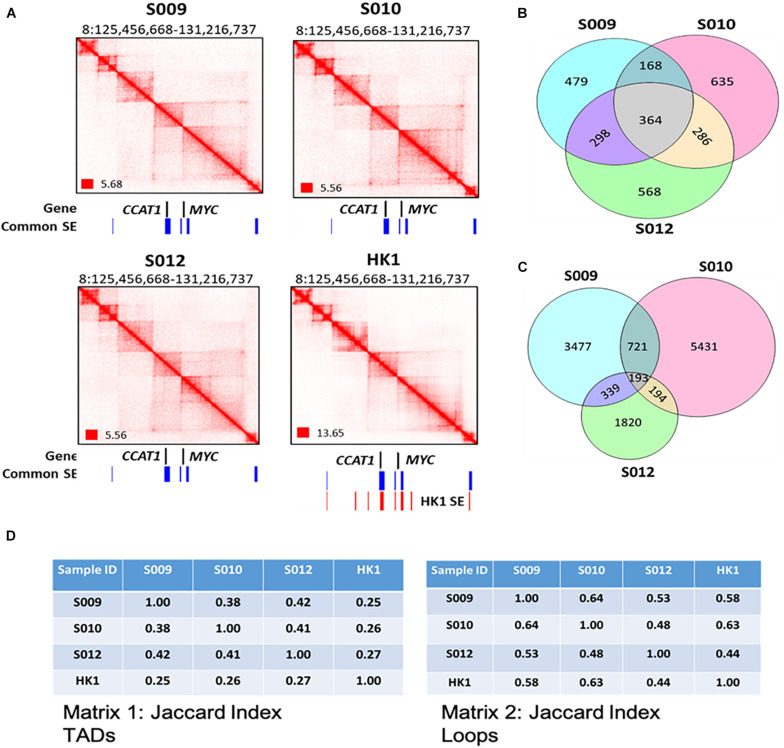
A genome-wide map of 3D genome organization in nasopharyngeal cancer. **(A)** Biop-C heatmaps can detect various conformational genomic features such as TADs, loops, and FIREs in S009, S010, and S012 at *CCAT1* and *MYC*. Coverage normalization was used to visualize the Biop-C and Hi-C heatmaps in Juicebox (Durand et al., 2016). Genes are indicated in black color. The “common super enhancers,” shown in blue color, indicate the super enhancers present in all three NPC cell lines—HK1, C66-1, and HNE1 cell lines. The super enhancers present in HK1 cell lines are indicated in red color. The super enhancer datasets are obtained from Ke et al. (2017). **(B)** Venn diagram showing the overlap of TAD boundaries between the patients inferred using the Arrowhead algorithm (Rao et al., 2014) at 10 kb resolution. **(C)** Venn diagram depicting the overlap of loops between the patients. The loops were called using the HiCCUPS algorithm (Rao et al., 2014) at 5, 10, and 25 kb resolution. The loops of the different resolutions were merged for this analysis. **(D)** Jaccard index. Matrix 1: Jaccard indices for TADs in NPC samples S009, S010, and S012 and cell line HK1; each cell in the matrix indicates the Jaccard index value for the column sample and row sample. Matrix 2: Jaccard indices for loops in NPC samples S009, S010, and S012 and cell line HK1 where each cell of the matrix indicates the Jaccard index value for the column and row sample.

Next, we examined the chromatin interactions around important oncogenes in NPC, such as *MYC* (Yu et al., 2003) (Figure 2A) and epidermal growth factor receptor (*EGFR*) (Supplementary Figure 2A) (Fujii et al., 2002; Chua et al., 2004; Ruan et al., 2011). *MYC* is overexpressed in 76% of the NPC patients. The patients with *MYC*-positive tumors had a longer disease-free period (Yu et al., 2003). *EGFR* is highly expressed in nearly 85% of NPC patients and associated with a significantly poorer prognosis in patients with advanced nasopharyngeal cancer than in patients without *EGFR* overexpression (Fujii et al., 2002; Chua et al., 2004). We found that *EGFR* is marked by three super enhancers in the HK1 cell line: two of these three SE are localized upstream, i.e., near the start transcription site, and the third one is located in an intron (Supplementary Figure 2B).

We further examined if there were any significant single nucleotide polymorphisms (SNPs) that were associated with loops in our NPC Biop-C samples. We observed an overlap of a loop anchor with a region at the CDKN2B(-AS1) locus in samples S009 and S010 (Supplementary Table 11), which was previously identified by a GWAS study on NPC in patients of Chinese descent (Bei et al., 2016).

**FIGURE 3:**
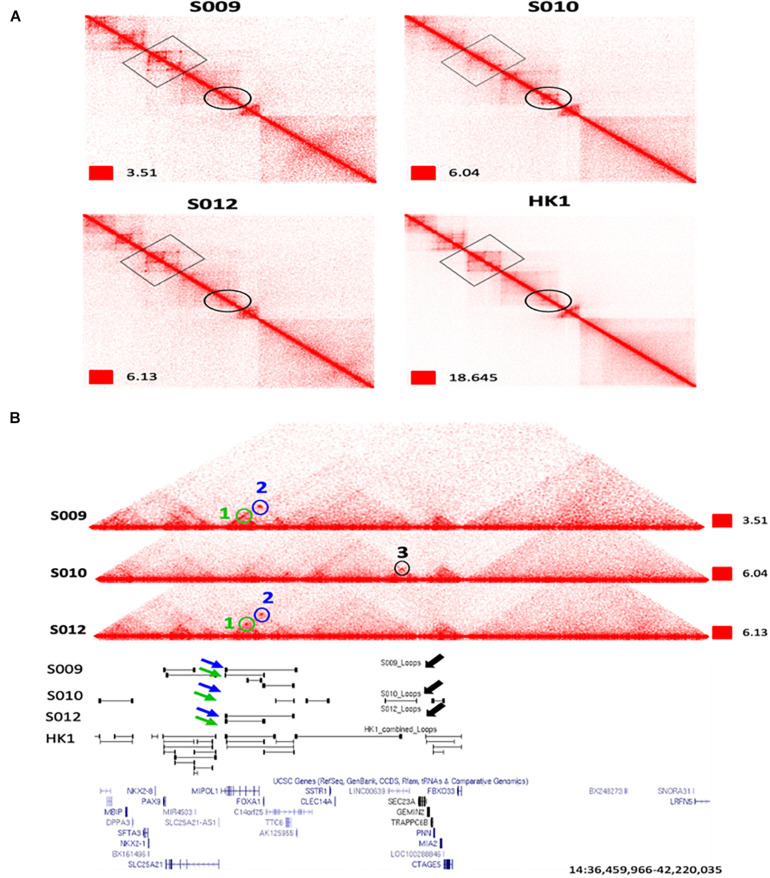
An example of extensive heterogeneity between patients in the chromatin interactome in nasopharyngeal cancer at the *FOXA1* and *MIPOL1* genes. **(A)** Zoomed view of Biop-C Juicebox-visualized heatmaps of S009, S010, and S012 showing patient heterogeneity in chromatin interaction at the *FOXA1* and *MIPOL1* genes. **(B)** UCSC genome browser (Kent et al., 2002) screenshots of genomic coordinates chr14: 36,459,966–42,220,035. Loops 1 and 2 are present in S009 and S012, while loop 3 is present only in S010. Loop 1, loop 2, and loop 3 are represented in green, blue, and black colors, respectively.

Additionally, for comparison with a typical Hi-C library, we examined the two replicates of HK1 NPC cell line that we generated by traditional *in situ* Hi-C (Arima kit) (Dixon et al., 2012; Rao et al., 2014). Visual inspection of coverage normalized Biop-C heatmaps of three NPC tissue samples, and Hi-C maps of the HK1 cell line showed that the libraries were largely similar to each other, although we also noted that certain loci contained differences suggesting patient heterogeneity which we explore further in the next section of this manuscript (Figures 3A,B and Supplementary Figures 3A,B).

To determine the reproducibility of our Biop-C method, we took one NPC patient sample, and cut it into half before performing the Biop-C method on the two separate halves. These are referred to as S024_R1 and S024_R2. We performed Juicer pipeline for calling TADs (247 in replicate 1 and 8 TADs in replicate 2) and loops (5,094 in replicate 1 and 2,199 in replicate 2) (Supplementary Figure 7A). We also called FIREs and found 1,707 FIREs in S024_R1 and 2,893 FIREs in S024_R2 (Supplementary Figure 7A). We used the HiCRep software to calculate the similarity between these replicates and found that 16 out of 24 chromosomes have high correlation (SCC scores above 0.8), while the other 8 chromosomes have SCC scores between 0.6 and 0.8, which shows that the two replicates are highly correlated (Supplementary Table 12). We also wanted to compare the similarity between S024 sample replicates with NPC cell line and a cell line from a different cancer. We found that the NPC samples showed higher similarity with HK1 (NPC cell line) as compared with T47D (breast cancer cell line) (Supplementary Figure 7B). We also did A/B compartment analysis for the two replicates and found a very high correlation between them (Pearson’s *r* = 0.95) (Supplementary Table 9 and Supplementary Figures 7C–E). These above results show that our Biop-C method is indeed reproducible.

Overall, our successful detection of TADs, loops, structural variants, A/B compartments, and FIREs suggests that our Biop-C method can generate high-quality genome-wide chromatin conformation maps from the solid tumor needle biopsy samples. Our evaluation using the S024 tumor cut in half and analyzed by two separate Biop-C libraries indicates that our Biop-C method is reproducible.

### Patient Heterogeneity in Chromatin Interactions

The question of patient heterogeneity is relatively unexplored in chromatin interaction analyses. In our previous research investigating chromatin interactions at the *TP53* and *MYC* loci, we observed that some chromatin interactions at MYC and TP53 could be detected in bone marrow and peripheral blood samples, but not all chromatin interactions that were observed in K562 cells were detected in clinical samples (Cao et al., 2017).

To investigate potential patient heterogeneity, we compared the TADs and loops between the Biop-C heatmaps of patients directly (Figures 2B,C) and using the Jaccard index. We calculated Jaccard’s similarity coefficient (Jaccard index, JI) to measure the overlap between the called TADs and loops in three Biop-C matrices. The resulting JI value indicates the fraction of shared TAD boundaries and loops between the patients. We observed that 38–42% of the TADs and 53–64% of loops are shared in the three patients (Figure 2D). We can conclude that the NPC samples show chromatin interaction heterogeneity. But since our samples are clinical samples, we cannot rule out the possibility that the patient chromatin interactome differences are due to the tumor heterogeneity and/or surrounding normal cells in the tumor.

Next, we examined individual specific chromatin interactions. We found that chromatin interactions for genomic locations *EGFR*, *PTPN1*, *DDIT4*, *MIR205HG*, *PDGFA*, *MALAT1*, *CAV2*, *NOTCH1*, *TEAD1*, *TP63*, *RUNX1*, *CCAT1*, *MYC*, and *YAP1* (Supplementary Figure 2A) are similar and *FOXA1*, *MIPOL1*, *SP4*, *SGCZ*, *MROH9*, *FMO1*, *FMO2*, *FMO1*, *FMO4*, and *FMO6P* gene are different (Figures 3A,B and Supplementary Figures 3A,B). In one example, we observed that two loops, i.e., loop 1 and loop 2 are present near *FOXA1* and *MIPOL1* genes in S009 and S012, which are thought to be tumor suppressors in nasopharyngeal cancer (Kwok et al., 2020; Leong et al., 2020). However, these loops were absent in S010 (Figures 3A,B). In another example, we observed a loop only in S009 and absent in S010 and S012 near miR383, which is considered an excellent diagnostic biomarker for head and neck cancers (Liu et al., 2019) (Supplementary Figure 3A). We also observed extensive chromatin looping near *MROH9*, *FMO1*, *FMO2*, *FMO1*, *FMO4*, and *FMO6P* locus in S009, which was absent in S010 and S012 (Supplementary Figure 3B).

Next, as we had observed patient-specific chromatin interactions, we investigated the tissue specificity of these chromatin interactions. Thus, we characterized the similarities and differences between the NPC landscape and other tissue types. We compared the Biop-C heatmaps and the Hi-C heatmap from HK1 with the previously published Hi-C heatmaps in human cell lines such as K562 (chronic myelogenous leukemia cell line) (Rao et al., 2014), HAP1 (near-haploid cell line) (Haarhuis et al., 2017), IMR90 (fetal lung fibroblast cell line) (Rao et al., 2014), KBM7 (chronic myelogenous leukemia) (Rao et al., 2014), HUVEC (human umbilical vein endothelial cell line) (Rao et al., 2014), RPE1 (retinal pigment epithelium cell line) (Darrow et al., 2016), GM12878 (lymphoblastoid cell line) (Rao et al., 2014), NHEK (normal human epidermal keratinocytes) (Rao et al., 2014), HeLa (human cervical carcinoma cell line) (Rao et al., 2014), HCT116 (colon cancer cell line) (Rao et al., 2014), and HMEC (mammary epithelial cell line) (Rao et al., 2014) (Supplementary Figures 2C–F).

We observed that the Hi-C heatmaps of K562, HAP1, IMR90, KBM7, HUVEC, and RPE1 show a similar pattern as the Biop-C heatmaps of S009, S010, and S012 and HK1 Hi-C heatmap for the genomic locations around *MYC* and *CCAT1*, but differences in the profile were observed in GM12878, NHEK, HeLa, HCT116, and HMEC (Supplementary Figure 2C). The genome organization at the *RUNX1* locus was similar between the Biop-C heatmaps of our patient in K562, HAP1, IMR90, RPE1, NHEK1, HeLa, HCT116, and HMEC but different in KBM7, HUVEC, and GM12878 (Supplementary Figure 2D). However, the genomic patterns for the *PTPN1* locus were found to be similar in all the Hi-C and Biop-C heatmaps of the patient (Supplementary Figure 2E). On the other hand, the genomic patterns for the *MALAT1* locus [*MALAT1* is known to promote cell proliferation in gastric cancer (Wang et al., 2014)] did not show any similarity and were different in all the Hi-C and Biop-C heatmaps (Supplementary Figure 2F). We conclude that certain chromatin interactions in nasopharyngeal cancer are common across tissue types (*PTPN1*) and certain regions are tissue specific (*MALAT1*).

The observation of patient heterogeneity and tissue specificity in TADs appears to contradict earlier observations that TADs are primarily conserved across different human cell types and possibly even across different species (Dixon et al., 2012; Jin et al., 2013; Rao et al., 2014). However, Sauerwald analyzed 137 Hi-C samples from nine studies and observed significant TAD variations across human cell and tissue types (Sauerwald et al., 2020), suggesting that while there are common TADs and loops, there are also TADs and loops that vary across patient samples and tissue types.

### Super Enhancers Are Associated With Frequently Interacting Regions and Loop to Genes

Super enhancers are regions of the DNA which enhances the transcription of target genes. These are comprised of a group of enhancers which are at close proximity to each other and are marked by high enrichment of H3K27ac histone modification (Pott and Lieb, 2015). In previous research, we and others have shown that super enhancers can regulate distant genes *via* long-range chromatin interactions (Cao et al., 2017; See et al., 2019). Moreover, FIREs have been reported to form at genomic regions also enriched by super enhancers (Schmitt et al., 2016). Consequently, we wished to understand the relationship between super enhancers and chromatin interactions in nasopharyngeal cancer.

**FIGURE 4:**
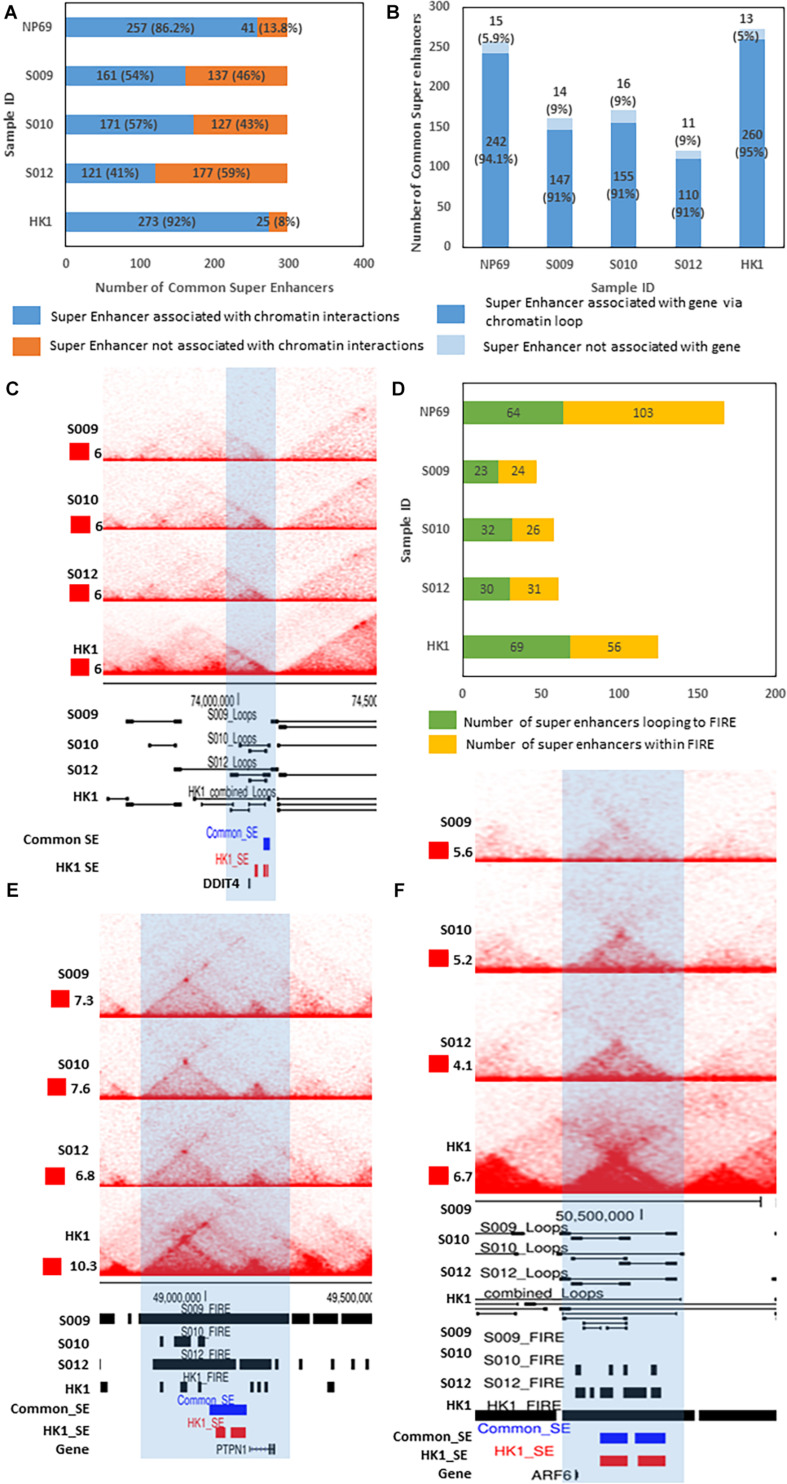
Association of super enhancers with chromatin interactions and genes. **(A)** Graph showing the number of common SE associated with chromatin interactions (blue) and the number of SE which are not associated with chromatin interactions (orange). **(B)** Graph showing the number of looping SE (chromatin interaction-associated SE) associated with distant genes *via* chromatin loops (dark blue) and the number of SE (chromatin interaction-associated SE) which do not link to distant genes *via* chromatin loops (light blue). **(C)** Biop-C heatmaps for samples S009, S010, and S012 and Hi-C heatmap for the cell line HK1 for the DDIT4 gene locus (zoomed-in view of genomic locus chr10:72974237–75095236) where a SE links to the gene through a chromatin loop. **(D)** Graph showing the number of SE associated with FIREs *via* chromatin loop (green) and the number of SE which are within FIRE (yellow). **(E)** Biop-C heatmaps for samples S009, S010, and S012 and Hi-C heatmap for the cell line HK1 (zoomed-in view of locus chr20: 48422029–49905948) showing a SE (blue: common SE, red: HK1 SE) and PTPN1 gene within the same FIRE. **(F)** Biop-C heatmaps for samples S009, S010, and S012 and Hi-C heatmap for HK1 (zoomed-in view of locus chr14: 49,497,144–51,494,634) showing a SE looping to FIRE at the ARF6 gene locus.

As the biopsy samples were too small for us to obtain both H3K27ac ChIP-Seq data as well as Biop-C data, we identified SE from NPC cell lines HK1, C666-1, and HNE1 using the ChIP-Seq data from Ke et al. (2017) (Supplementary Table 13) and we found that 298 SE were common in all the three cell lines. We reasoned that these “common” super enhancers that are present in all cell lines examined will most likely also be present in the patient samples examined and hence used them for downstream analysis. We then associated these common super enhancers with chromatin loops obtained from the Biop-C data of the patient samples as well as from the Hi-C data of HK1 and NP-69 cell lines. As a result, we found that these SE are highly associated with chromatin loops. In samples S009, S010, and S012, we found 54, 57, and 41% (respectively) SE which were associated with chromatin loop anchors within 15 kb distance (“looping SE”) (Figure 4A).

We further looked for associations between these looping SE and genes (Supplementary Figure 8A). We observed that more than 90% of looping SE loop to distant genes (Supplementary Figure 8A). In S009, 95% of looping SE are linked to distant genes *via* these loops. In S010 as well as in S012, 91% of looping SE are linked to distant genes (Figure 4B and Supplementary Table 13). We also associated these SE to chromatin loops in cell line HK1. We found that 92% of common super enhancers are associated with chromatin loops in HK1 and 91% of these looping SE are linked to distant genes (Figures 4A,B and Supplementary Table 15). For example, the *DDIT4* gene (one of the top ranked SE-associated gene in the HK1 cell line) (Ke et al., 2017) is also associated with a distant SE in patient samples S010 and S012 and NPC cell line HK1 (Figure 4C). We repeated the above analysis with cell line NP-69 and found that 86.2% of SE are associated with chromatin interactions and 94.1% of looping SE are linked to distal genes (Figures 4A,B). This coincides with our previous results that the number of chromatin loops lost is more than the acquired loops in NPC, and hence, we can see more SE association with loops in NP-69 as compared with NPC samples.

We also repeated this analysis with common enhancers within cell lines HK1, C661, and HNE1. In samples S009, S010, and S012, the association of enhancers with chromatin loops was 24, 29, and 18%, respectively, and about 60% (63, 65, and 64% in S009, S010, and S012, respectively) of these chromatin interaction-associated enhancers loop to distal genes (Supplementary Figures 8D,E). We also repeated this analysis with cell lines HK1 and NP-69 and found that 44.3 and 43.8% SE are associated with loops, respectively, and about 56% (56.2% in NP-69 and 55.6% in HK1) of these chromatin interaction-associated enhancers loop to distal genes (Supplementary Figures 8D,E). From these results, we conclude that the association of enhancers with chromatin loops is much less as compared with SE in NPC patients. In NP-69, the association of enhancers with chromatin loops is higher compared with NPC samples but less as compared with SE in NP-69.

Next, we categorized common SE based on their proximity to a gene: the ones which were at close proximity (less than 15 kb from a gene) to a gene were called “proximal” SE and the ones which are away from the gene and associated *via* chromatin loops were called distal SE. Out of 298 common SE tested, we found 27 proximal SE in all the Biop-C samples and 147 distal SE in S009, 155 distal SE in S010, 110 distal SE in S012, and 260 distal SE in HK1. We observed that 48 genes were associated with proximal SE (Supplementary Table 14), while 356 genes in S009, 421 genes in S010, 291 in S012, and 944 genes in HK1 were associated with distal SE (Supplementary Table 16). There were also some genes that have both proximal and distal SE: in S009, we found 10 genes; in S010 as well as S012, we found 6 genes, and in HK1, we found 18 such genes (Supplementary Table 16). For example, the *MACF1* gene in all the three NPC samples as well as HK1 cell line has distal as well as proximal SE (Supplementary Table 16). Gene set overrepresentation analyses (GSOA) on the set of genes associated with SE showed significant overrepresentation of Biological Process Gene Ontology (GO) terms such as metabolic process and protein modification/phosphorylation and regulation (Supplementary Figure 9).

Subsequently, we wanted to associate these SE with FIREs called (Supplementary Table 10) from the Biop-C data from patient samples as well as HK1 and NP-69 Hi-C data. We looked for two types of associations between SE and FIREs: SE within FIRE and SE that loop to FIRE (Supplementary Figures 8B,C). We could recognize 24 common SE (8% of SE) in S009, 26 (9% of SE) in S010, 31 (11% of SE) in S012, 56 (19% of SE) in HK1, and 103 (35% of SE) in NP-69 which are within a FIRE (Figure 4D). Upon combining the chromatin loops data with FIRE calling, we found 23 (8% of SE) in S009, 32 (11% of SE) in sample S010, 30 (10% of SE) in S012, 69 (23% of SE) in HK1, and 64 (22% of SE) in NP-69 cell line that loops to a FIRE (Figure 4D and Supplementary Table 15). We also found some examples of SE associated with genes *via* FIRE. For example, SE which falls within the same FIRE as the gene *PTPN1* (Figure 4E) and SE which loops to a distant FIRE containing the *ARF6* gene whose overexpression can be correlated with metastasis and invasion in several cancers (Li et al., 2017) (Figure 4F).

We also repeated this analysis with enhancers and found 512 (3.8% of total enhancers), 270 (2% of total enhancers), and 524 (4% of total enhancers) enhancers within FIRE in S009, S010, and S012, respectively, and 242 (1.8% of total enhancers), 285 (2.1% of total enhancers), 189 (1.4% of total enhancers) enhancers looping to FIRE in samples S009, S010, and S012 (Supplementary Figure 8F). In the cell lines, we found 783 (5.8% of total enhancers) and 868 (6.5% of total enhancers) enhancers within FIRE in HK1 and NP-69, respectively; 539 (4% of total enhancers) and 868 (6.4% of total enhancers) enhancers looped to FIRE in HK1 and NP-69 (Supplementary Figure 8F). Based on these findings, we can conclude that SE are associated with FIREs in NPC. However, the SE association with FIRE is higher in NP-69 as compared with the NPC patient sample.

### THZ1 Treatment Leads to Loss of Specific Super Enhancer– and Typical Enhancer–Promoter Chromatin Interactions at Key Oncogenic Loci

To investigate the functional significance of SE–promoter loops in NPC, we examined changes in the chromatin interactome *via* conventional *in situ* Hi-C, following the treatment of HK1 cells with the CDK7 inhibitor THZ1. In several cancers, THZ1 treatment leads to decreased cell viability (Chipumuro et al., 2014; Kwiatkowski et al., 2014). The binding of THZ1 to CDK7 was previously shown to lead to inhibited CDK7-mediated phosphorylation of RNA Pol II, which coincides with a loss of transcription factor binding at distal sites with high H3K27ac (Sampathi et al., 2019), representing putative super and typical enhancer loci (Hnisz et al., 2013). SE-associated genes are exceptionally sensitive to perturbation by THZ1 treatment (Ke et al., 2017). However, alterations in the chromatin interactome upon CDK7 inhibition in NPC cells have not been previously explored.

**FIGURE 5:**
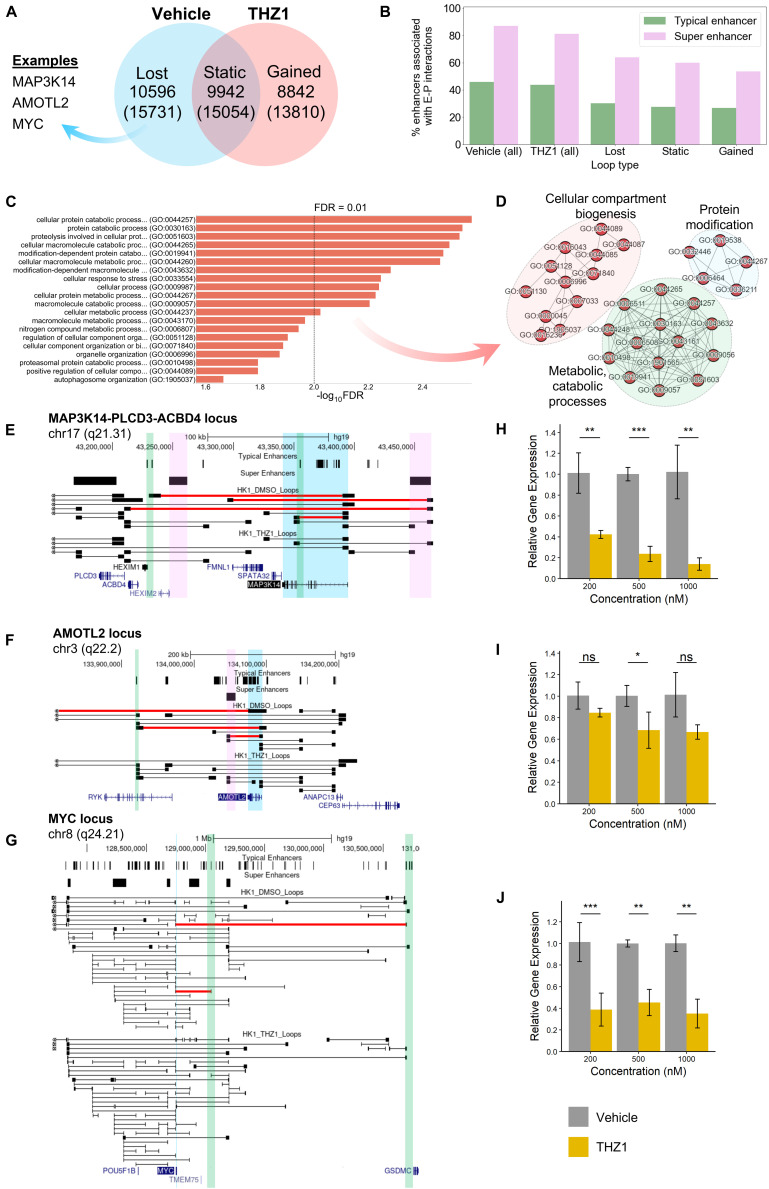
**(A)** Functional analysis of HK1 chromatin interactome following treatment with CDK7 inhibitor THZ1. **(A)** Venn diagram representing the number of loops lost after THZ1 treatment compared with DMSO (vehicle) treatment, unchanged between treatments, and gained after treatment. The number of genes with promoter regions within 15 kb of the respective loop anchors is shown in the parentheses below. **(B)** Anchor occupancy characteristics of super enhancers and typical enhancers predicted from H3K27ac ChIP-Seq. A higher percentage of super enhancers is associated with enhancer–promoter loops, compared with typical enhancers. **(C)** Top 20 overrepresented GO-Slim Biological Process terms by false discovery rate (FDR), derived from the set of protein-coding genes with net lost loops following THZ1 treatment. **(D)** Network representation of predominantly enriched GO term clusters. **(E–G)** Looping structure of MAP3K14, AMOTL2, and MYC loci. Lost loops following THZ1 treatment are shown in red. Gene of interest is highlighted in blue. Select super enhancers with lost loops to promoters are highlighted in pink. Select typical enhancers with lost loops to promoters are highlighted in green. **(H–J)** RT-qPCR results for MAP3K14, AMOTL2, and MYC, respectively, following 24 h of vehicle or THZ1 treatment (*p ≤ 0.05, **p ≤ 0.01, ***p ≤ 0.001).

To investigate this, we performed *in situ* Hi-C on THZ1 and DMSO (vehicle)-treated HK1 cells at 500 nM concentration for 24 h. THZ1 treatment led to clear changes in the chromatin interactome: we observed that 51.6% (10,596) of the loops present under vehicle treatment were lost following THZ1 treatment, whereas 48.4% (9,942) were conserved. Interestingly, 8,842 loops were gained following THZ1 treatment (Figure 5A).

As with NPC Biop-C samples, we observed that approximately 80% of all super enhancers identified by HK1 H3K27ac ChIP-Seq were associated with long-range enhancer–promoter interactions, compared with only 40% of typical enhancers, regardless of vehicle or THZ1 treatment (Figure 5B and Supplementary Table 16). Gene set overrepresentation analyses on the genes with net lost loops revealed a significant enrichment of GO terms associated with cellular stress response, compartment/organelle biogenesis, metabolic/catabolic processes, and protein modification (Figures 5C,D). Clusters relating to metabolic processes and protein modification were also observed for the SE-associated genes identified in the three NPC Biop-C samples (Supplementary Figure 8), further suggesting that SE are a common dependency-mediating expression at the pathway level.

Subsequently, to determine if lost loops to super or typical enhancers could explain THZ1-mediated loss of viability and gene expression, we performed a close examination of the looping behavior in three candidate genes, namely, *MAP3K14*, *AMOTL2*, and *MYC*, which are known to be involved in NPC pathogenesis (Figures 5E–G). At the *MAP3K14* and *AMOTL2* loci, we observed the loss of super and typical enhancer-associated loops to these promoters, whereas at the *MYC* locus, we only observed the loss of typical enhancer-associated loops. *Via* RT-qPCR, we additionally observed decreased expression of all three genes following THZ1 treatment, compared with vehicle (Figures 5H–J).

We also compared the A/B compartments of HK1 (control) and HK1–THZ1 treated and found a high correlation between them (Pearson’s *r* = 0.95) suggesting that the A/B compartments remain conserved upon THZ1 treatment (Supplementary Figure 10).

As a whole, these results suggest that THZ1 treatment leads to specific perturbations of the chromatin interactome. Some of the lost loops correspond to specific SE– and typical enhancer–promoter interactions that are involved in the control of expression at these loci. THZ1 treatment leads to downregulation of expression at several of these loci, further suggesting the regulatory role of these SE- and typical enhancer-associated loops.

## Conclusion

Taken together, our new Biop-C method is suitable for interrogating needle biopsy patient samples and, more generally, situations of limited tumor sampling when surgical biopsies may be technically difficult. Using Biop-C, we examined chromatin interactions in three nasopharyngeal cancer patient samples, which allowed us to identify super enhancers associated with FIREs and which loop to important oncogenes. We also demonstrated patient heterogeneity in chromatin interactions in these patient samples, as well as tissue specificity. Hi-C libraries from an NPC cell line, HK1, showed differences compared with chromatin interactions in the patient samples. These differences could arise due to different subtypes of NPC.

Upon comparison with near-normal nasopharynx cell line NP-69, we found that while there were some loops that were similar between NPC samples and NP-69, there were also loops that were different, suggesting that there may be NPC-specific loops that could potentially regulate NPC oncogenes. To test the reproducibility of our method, we also performed Biop-C on two replicates of the same patient sample and found high correlation between the two replicates in most of the chromosomes (16 out of 24). Upon comparison with cell lines, we found that NPC patient replicates were highly correlated with the NPC cell line HK1 as compared with the breast cancer cell line T57D. We also observed that SE are much more associated with chromatin interactions and FIREs as compared with enhancers; however, these associations are higher in NP-69 as compared with NPC samples, which is consistent with our previous findings that more loops are lost in NPC as compared with those acquired. Our results indicate the necessity of interrogating chromatin interactions in actual patient cancers, which Biop-C enables. Our results demonstrate the utility and importance of Biop-C as a method for understanding cancer 3D genome organization. Additionally, our results suggest that THZ1 may be able to modulate chromatin interactions. In the future, we anticipate that the versatility of Biop-C will also allow us to interrogate perturbations of chromatin gene regulation in patients undergoing therapeutic interventions.

## Data Availability Statement

The datasets of Hi-C are available in GEO under accession number GSE166570 (reviewer token: yhizaucglrghlmp).

## Ethics Statement

The studies involving human participants were reviewed and approved by Institute Review Board, number 2018/00947-SRF0002. The patients/participants provided their written informed consent to participate in this study.

## Author Contributions

SA and MF conceived the research idea. MF, SA, and RC contributed to the study design. SA and MF developed the Biop-C method. SA, HJ, and LC prepared Biop-C and HiC libraries. SA and RC performed Hi-C analysis of the Biop-C and Hi-C samples. SA performed manual curation of the Hi-C data. RC performed FIRE calling, Structural Variants calling, A/B compartment analysis, replicate comparison, and ChIP-Seq analysis on published data to identify SEs and their looping patterns in NPC. BW performed Fithic2 calling, enhancer promoter association, loops comparison between NPC and NP-69 samples, and Gene ontology. SA and CK performed THZ1 treatment. BW performed RT-qPCR and data analysis. XY, JT, W-QC, and BG provided NPC clinical samples. SA, RC, and MF reviewed the data.

## Conflict of Interest

MF declares two patents on methodologies related to ChIA-PET. The remaining authors declare that the research was conducted in the absence of any commercial or financial relationships that could be construed as a potential conflict of interest.

## Publisher’s Note

All claims expressed in this article are solely those of the authors and do not necessarily represent those of their affiliated organizations, or those of the publisher, the editors and the reviewers. Any product that may be evaluated in this article, or claim that may be made by its manufacturer, is not guaranteed or endorsed by the publisher.

## Footnotes

1
https://github.com/4dn-dcic/hic2cool
