# Supplementary material for: Profiling of 3D Genome Organization in Nasopharyngeal Cancer Needle Biopsy Patient Samples by a Modified Hi-C Approach
Source: Front Genet. 2021 Sep 3;12:673530. doi: 10.3389/fgene.2021.673530 (PMC8446523; doi:10.3389/fgene.2021.673530)
Supplement: Supplementary file 1 [file Data_Sheet_1.PDF]

Supplementary Materials

Profiling of 3D genome organization in Nasopharyngeal Cancer needle biopsy patient samples by a modified Hi-C approach

Sambhavi Animesh [1†], Ruchi Choudhary [1, 2 †], Bertrand Wong Jern Han[2], Charlotte Koh Tze Jia[2], Xin Yi Ng [3], Joshua Kai Xun Tay [4], Wan-Qin Chong [3], Han Jian[1,2], Leilei Chen [1,6], Boon Cher Goh [1, 3, 5], Melissa Jane Fullwood [1, 2]*

[1] Cancer Science Institute of Singapore, Centre for Translational Medicine, National University of Singapore, Singapore
[2]School of Biological Sciences, Nanyang Technological University, Singapore
[3]Department of Haematology-Oncology, National University Cancer Institute of Singapore, National University Health System, Singapore
[4]Department of Otolaryngology - Head & Neck Surgery, National University Hospital, Singapore
[5]Department of Pharmacology, Yong Loo Lin School of Medicine, National University Health System, Singapore,
[6]Department of Anatomy, Yong Loo Lin School of Medicine, National University of Singapore, Singapore

[†]These authors have contributed equally to the work
**Corresponding author*
Corresponding author contact: Melissa Jane Fullwood, mfullwood@ntu.edu.sg

Contents:

1. **Supplementary Table Legends**
2. **Supplementary Figure Legends**
3. **Supplementary Tables**
4. **Supplementary Figures**
5. **Supplementary References**

Supplementary Table Legends

Table S1. Comparison between different low input Hi-C method.

Table S2. Clinical Details of nasopharyngeal cancer sample used in the manuscript.

Table S3. Juicer-reported statistics of Hi-C libraries in this manuscript (Excel file) Sheet QC: Contains information about the sequenced read. Column A: sample ID, Column B: number of total sequenced reads, Column C: number of reads that could be successfully aligned. Column D: number of unique reads, Column E: PCR duplicates, F: Optical duplicates, G: number of HiC contacts, H: number of reads below MAPQ value 30, I: number of intra-fragment reads. Sheet Interactions contains information about the type of interaction that were recognized by the software. Column A: sample ID, B: number of inter-chromosomal interactions, C: number of intra-chromosomal interactions, D: short range interactions, E: long range interactions.

Table S4. TADs called by Arrowhead from Hi-C libraries in this manuscript (Excel file). Sheet S009: TADs called from NPC sample S009, Sheet S010: TADs called from NPC sample S010, Sheet S012: TADs called from NPC sample S012 , Sheet HK1: TADs called from cell line HK1 (both replicates merged), Sheet HK1_R1: TADs called from HK1 replicate 1, Sheet HK1_R2: TADs called from HK1 replicate 2, Sheet NP69: TADs called from NP-69, Sheet S024_R1: TADs called from S024 replicate 1 and Sheet S024_R2: TADs called from S024 replicate 2. In each sheet, Column A-C indicates the genomic location of the TADs called. (A: chromosome no, B: start, C: end)

Table S5. Chromatin loops called by HiCCUPS from Hi-C libraries in this manuscript (Excel file). Sheet S009: Chromatin loops called from sample S009, Sheet S010: Chromatin loops called from NPC sample S010, Sheet S012: Chromatin loops called from sample S012 and Sheet HK1: Chromatin loops called from cell line HK1 (both replicates merged), Sheet HK1_R1: Loops called from HK1 replicate 1, Sheet HK1_R2: Loops called from HK1 replicate 2, Sheet NP69: Loops called from NP-69, Sheet S024_R1: Loops called from S024 replicate 1 and Sheet S024_R2: Loops called from S024 replicate 2. In each sheet, Column A-C: genomic location of anchor 1 for each loop (A: chromosome no, B: start, C: end). D-F: genomic location of anchor 2 for each loop (D: chromosome no, E: start, F: end).

Table S6. List of loops mediating SE- and typical enhancer-promoter interactions in NPC samples (Excel file). Sheet 009_SE: List of loops involved in SE-promoter interactions in NPC patient sample S009. Sheet 0010_SE: List of loops involved in SE-promoter interactions in NPC patient sample S0010. Sheet 0012_SE: List of loops involved in SE-promoter interactions in NPC patient sample S0012. Sheet 009_TE: List of loops involved in typical enhancer-promoter interactions in NPC patient sample S009. Sheet 0010_TE: List of loops involved in typical enhancer-promoter in NPC patient sample S0010. Sheet 0012_TE: List of loops involved in typical enhancer-promoter interactions in NPC patient sample S0012. In each sheet, Column A-D: genomic co-ordinates of loops (A: chromosome no., B: gene anchor position, C: left anchor centroid, D: right anchor centroid), Column E,F: HiCCUPS significance metrics for each called loop (E: fdrBL, F: fdrDonut), G: genes with promoter proximal to given anchor, J:

enhancers promoter to other anchor, I: number of genes with promoter proximal to given anchor, J: number of enhancers proximal to other anchor.

Table S7. Summary count of significant chromatin interactions called by FitHiC2 from NPC Biop-C libraries in this manuscript (Excel file). Comprehensive list of interactions is available on request.

Table S8. Translocation called from Biop-C samples and NPC cell lines (Excel file). Sheet S009: Translocations identified in sample S009, Sheet S012: Translocations identified in S012, Sheet HK1: Translocations identified in HK1, Sheet NP69: Translocations identified in sample NP-69. Column A-B: Coordinates of region one, Column C: breakpoint for region one, Column D-E: Coordinates of region two, Column F: Breakpoint of region 2, Column G: p-value.

Table S9. A/B compartment analysis. Sheet 1: Column A-C: genomic coordinates of the bins (A: chromosome no, B: start, C: end), Column D: Eigen values for S009, Column E: Eigen values for S010, Column F: Eigen values for S012 and Column G: Eigen values for NP-69. Sheet 2: Column A-C: genomic coordinates of the bins (A: chromosome no, B: start, C: end), Column D: Eigen values for S024 replicate1, Column E: Eigen values for S024 replicate2. Sheet 3: Column A-C: genomic coordinates of the bins (A: chromosome no, B: start, C: end), Column D: Eigen values for HK1 DMSO (control), Column E: Eigen value for HK1 THZ1 treated.

Table S10. FIREs called by FIREcaller from HiC libraries in this manuscript. (Excel file). Sheet S009: FIREs called from sample S009, Sheet S010: FIREs called from sample S010, Sheet S012: FIREs called from sample S012, Sheet HK1: FIREs called from cell line HK1 (both replicates merged), Sheet HK1_R1: FIREs called from HK1 replicate 1, Sheet HK1_R2: FIREs called from HK1 replicate 2, Sheet NP69: FIREs called from NP-69, Sheet S024_R1: FIREs called from S024 replicate 1 and Sheet S024_R2: FIREs called from S024 replicate 2. In each sheet, Column A-C: Genomic coordinates of FIRE (A: chromosome no, B: start, C: end)

Table S11. List of loops proximal to SNP in NPC samples (Excel file). S009_SNP: SNPs proximal to loop anchors in NPC patient sample S009. S010_SNP: SNPs proximal to loop anchors in NPC patient sample S010. No SNP-anchor associations were found for patient sample S012. In each sheet, Column A-D: genomic co-ordinates of loops (A: chromosome no., B: SNP anchor position, C: left anchor centroid, D: right anchor centroid), Column E,F: HiCCUPS significance metrics for each called loop (E: fdrBL, F: fdrDonut), G: SNP identity number, H: SNP coordinate, I: mapped gene(s), J: SNP p-value, H: SNP GWAS study accession.

Table 12. SCC scores from Hicrep for S24 replicate 1 and replicate 2 comparison. Column A: Chromosome number, Column B: SCC score.

Table S13. List of H3K27ac ChIP-seq libraries used for SE calling (Excel file). Column A: type of cell line. C: ChIP-seq antibody (h3k27ac or none). C: library accession number. D: file format. E: file accession number. Sheet S009: List of SE and associated genes in NPC sample S009, Sheet S010: list of SE and and associated gene in sample S010, Sheet S012: list of SE and associated gene in sample S012 and Sheet HK1: list of SE and associated genes in cell line HK1.In each sheet, Column A-C: genomic coordinates of super enhancer (A: chromosome no, B: start, C: end). Column D-F: genomic coordinates of 1st loop anchor (D: chromosome no, E:

start, F: end). Column G-I: genomic coordinates of 2nd loop anchor (G: chromosome no, H: start, I: end). Column J: Gene name.

Table S14. List of genes which are associated with both distant and proximal super enhancers (Excel file). Sheet Proximal gene: Contains list of genes which are proximal to SE common in all the three NPC cell lines. Sheet S009: list of genes which are associated with proximal as well as distant super enhancers in NPC patient sample S009. Sheet S010: list of genes which are associated with both proximal as well as distant super enhancers in sample S010, Sheet S012: list of genes which are associated with both proximal as well as distal super enhancers in sample S012. Sheet HK1: list of genes which are associated with both proximal as well as distal super enhancers in NPC cell line HK1.

Table S15. List of SEs associated with FIREs via chromatin loops (Excel file). Sheet S009: List of SE associated with FIRE via chromatin loops in NPC sample S009, Sheet S010: List of SE associated with FIRE via chromatin loops in NPC sample S010, Sheet S012: List of SE associated with FIRE via chromatin loops in NPC sample S012. In each sheet, Column A-C: genomic co-ordinates for super enhancer (A: chromosome no, B: start, C: end), Column D-F: genomic co-ordinates for loop anchor 1 (D: chromosome no, E: start, F: end), Column G-I: genomic co-ordinates for loop anchor 2 (G: chromosome no, H: start, I: end), Column J-L: genomic co-ordinates for FIRE (J: chromosome no, K: start, L: end).

Table S16. List of loops mediating SE- and typical enhancer-promoter interactions in HK1 samples treated with either DMSO or THZ1 (Excel file). Lost_SE: List of lost loops following THZ1 treatment involved in SE-promoter interactions. Static_SE: List of unchanged loops following THZ1 treatment involved in SE-promoter interactions. Gained_SE: List of gained loops following THZ1 treatment involved in SE-promoter interactions. Lost_TE: List of lost loops following THZ1 treatment involved in typical enhancer-promoter interactions. Static_TE: List of unchanged loops following THZ1 treatment involved in typical enhancer-promoter interactions. Gained_TE: List of gained loops following THZ1 treatment involved in typical enhancer-promoter interactions. In each sheet, Column A-D: genomic co-ordinates of loops (A: chromosome no., B: gene anchor position, C: left anchor centroid, D: right anchor centroid), Column E, F: HiCCUPS significance metrics for each called loop (E: fdrBL, F: fdrDonut), G: genes with promoter proximal to given anchor, F: enhancers promoter to other anchor, H: number of genes with promoter proximal to given anchor, I: number of enhancers proximal to other anchor.

Supplementary Figure Legends

Fig S1: Biop-C, a modified *in situ* Hi-C method**.** Representative images of needle biopsy samples of nasopharyngeal cancer.

Fig S2 A: A Genome-wide Map of 3D Genome Organization in Nasopharyngeal Cancer**.** Biop-C heat maps can detect various conformational genomic features such as TADs, Loops, and FIREs in S009, S010, and S012 at key oncogenes. Coverage normalization was used to visualize the Biop-C and Hi-C heatmaps in Juicebox (Rao et al., 2014).The 3 dimensional genome organization at key oncogenes such as *EGFR, PTPN1, DDIT4, MIR205HG, PDGFA, MALAT1, CAV2, NOTCH1, TEAD1, TP63, RUNX1,* and *YAP1* of nasopharyngeal cancer. Genes are indicated in black color. The "common superenhancers", shown in blue color, indicate the super enhancers present in all three NPC cell lines- HK1, C66-1, and HNE1 cell lines. The super enhancers present in HK1 cell lines are indicated in red color. The super enhancer datasets are obtained from Ke et al. 2017 (Ke et al., 2017).

Fig S2 B: UCSC genome browser screenshot showing the location of super enhancers and chromatin looping near *EGFR* gene (chr7:54,820,528-55,501,570).

Fig S2 C-F: Juicebox images of the (C) *CCAT1* and *MYC* (chr8:125,456,668-131,216,737) (D) *RUNX1* (chr21:33,410,812-39,170,881) (E) *PTPN1* (chr20:46,366,857-52,126,926) (F) *MALAT1* (chr11:62,487,511-68,247,580) regions from published Hi-C libraries from human cell lines such as K562 (chronic myelogenous leukemia cell line) (Rao et al., 2014), HAP1 (near-haploid cell line) (Haarhuis et al., 2017), IMR90 (fetal lung fibroblast cell line) (Rao et al., 2014), KBM7 (chronic myelogenous leukemia) (Rao et al., 2014), HUVEC (human umbilical vein endothelial cell line) (Rao et al., 2014), RPE1 (retinal pigment epithelium cell line) (Darrow et al., 2016), GM12878 (lymphoblastoid cell line) (Rao et al., 2014), NHEK (normal human epidermal keratinocytes) (Rao et al., 2014), HeLa (human cervical carcinoma cell line) (Rao et al., 2014), HCT116 (colon cancer cell line) (Rao et al., 2017), HMEC (mammary epithelial cell line) (Rao et al., 2014). For comparison, we also show the Biop-C heatmaps from this paper's patient sample S010. Coverage normalization was used to generate heatmaps.

Fig S3A: One example of extensive heterogeneity between patients in the chromatin interactome in Nasopharyngeal cancer at the miR383 gene locus. Zoomed-in view of BiopC heat map of S009, S010, and S012 at *SGCZ* gene and miR383 (top panel). UCSC genome browser screenshots of genomic coordinate chr8:11,979,966-17,740,035. There is a loop present near miR383 only in S009 and absent in S010 and S012 (bottom panel).

Fig S3B. One example of extensive heterogeneity between patients in the chromatin interactome in Nasopharyngeal cancer at the *FMO* locus.Zoomed-in view of BiopC heat map of S009, S010, and S012 at *MROH9, FMO1, FMO2, FMO1, FMO4,* and *FMO6P* gene (top panel). UCSC genome browser screenshots of genomic coordinate chr1:168,314,966-174,075,035. There is extensive chromatin looping near *MROH9, FMO1, FMO2, FMO1, FMO4,* and *FMO6P* locus in S009 only (bottom panel).

Fig S4. (A) Table of statistics of NP-69. #Hi-C contacts indicates number of mapped/valid junction reads of each library. #TAD indicates the number of TADs called at 10kb resolution.

#Loops indicates the total number of loops called at 5kb, 10 kb, and 25 kb resolution and then merged. # FIREs indicates the number of FIREs called at 10kb resolution. (B) Venn diagram representing the number of loops that are specific to NPC, similar between NPC and NP-69, and specific to NP-69. The number of genes with promoter regions within 15kb of the respective loop anchors is shown in the parentheses below.

Fig S5. Structural variant calling using Biop-C and Hi-C data (A) Whole genome Biop-C heatmap of sample S009 indicating identified translocations (blue circle) (B) Whole genome Biop-C heatmap of sample S010 (no translocations were identified in the sample) (C) Whole genome Biop-C heatmap of sample S012 indicating identified translocations (blue circle) (D) Whole genome Biop-C heatmap of cell line HK1 indicating identified translocations (blue circle) (E) Whole genome Biop-C heatmap of cell line NP69 indicating identified translocations (blue circle).

Fig S6. A/B compartment analysis. (A) Heatmap of sample S009 for chr 3 indicating A (active) B (inactive) domain. The graph below the heatmap indicates eigen value for each bin in chr 3. (B) Heatmap of sample S010 for chr 3 indicating A (active) B (inactive) domain. The graph below the heatmap indicates eigen value for each bin in chr 3. (C) Heatmap of sample S012 for chr 3 indicating A (active) B (inactive) domain. The graph below the heatmap indicates eigen value for each bin in chr 3. (D) Heatmap of sample NP-69 for chr 3 indicating A (active) B (inactive) domain. The graph below the heatmap indicates eigen value for each bin in chr 3. (E) Scatter plot of eigen values between S009 and S010. (F) Scatter plot of eigen values between S009 and S012. (G) Scatter plot of eigen values between S010 and S012. (H) Scatter plot of eigen values between S009 and NP-69. (I) Scatter plot of eigen values between S010 and NP-69. (I) Scatter plot of eigen values between S012 and NP-69.

Fig S7. (A) Table of statistics of S024 replicates R1 and R2. #Hi-C contacts indicates number of mapped/valid junction reads of each library. #TAD indicates the number of TADs called at 10kb resolution. #Loops indicates the total number of loops called at 5kb, 10 kb, and 25 kb resolution and then merged. # FiREs indicates the number of FIREs called at 10kb resolution. (B) Graph showing SCC scores from hicrep analysis of samples HK1 R1 and R2, S024 R1 and R2, T47D R1 and R2, HK1 R1 and S024 R1, HK1 R1 and S024 R2, HK1 R2 and S024 R1, HK1R2 and S024 R2, HK1 R1 and T47D R1, HK1 R1 T47 R2, HK1 R2 and T47 R1, HK1 R2 and T47D R2, S024 R2 and T47D R1, S024 R2 and T47D R2, S024 R1 and T47D R1, S024 R1 and T47D R2. (C-D)) Heatmap of sample S024 R1 and R2 for chr 2 indicating A (active) B (inactive) domain. The graph below the heatmap indicates eigen value for each bin in chr 2. (E) Scatter plot of eigen values between S024_R1 and S024_R2.

Fig S8. Chromatin interactions associated with enhancers. (A) Graphical representation of chromatin loop between SE/enhancer (blue box) and a distal gene (green arrow). The encircled region in the heatmap shows the chromatin loop (B) Graphical representation of SE/enhancer within FIRE (red box). Encircled region in the heatmap respresents FIRE. (C) Graphical representation of SE/enhancer looping to FIRE. The encircled region in the heatmap shows the FIRE. (D) Graph showing the number of enhancers associated with chromatin interactions (blue) and number of enhancers which are not associated with chromatin interactions (orange). (E) Graph showing number of enhancers (chromatin interaction associated enhancers) associated with distant genes via chromatin loops (dark

blue) and number of enhancers (chromatin interaction associated enhancers) which do not link to distant genes via chromatin loops (light blue). (F) Graph showing the number of enhancers associated with FIREs via chromatin loop (green) and number of enhancers which are within FIRE (yellow).

Fig S9A. Top 20 significantly enriched Biological Process GO terms, obtained via gene set overrepresentation analysis on the list of SE-associated genes common to all 3 NPC Biop-C samples (S009, S010, S012).

Fig S9B. Corresponding clusters of enriched GO terms by Lin Semantic Similarity (LSS) measure. Clusters of size 3 or larger are shown.

Fig S10. (A) Heatmap of HK1 DMSO treated (control) for chr 2 indicating A (active) B (inactive) domain. The graph below the heatmap indicates eigen value for each bin in chr 2. (B) Heatmap of HK1 THZ1 treated for chr 2 indicating A (active) B (inactive) domain. The graph below the heatmap indicates eigen value for each bin in chr 2. (C) Scatter plot of eigen values between HK1 DMSO (control) and HK1 THZ1 treated.

__**Supplementary Tables**__

Table S1

	as small-scale *in situ* Hi-C (sisHi-C)(Du et al., 2017)	easy Hi-C (Lu et al., 2020)	Low-C (Díaz et al., 2018)	Biop-C
Sample	Embryos and cell lines	Cell line and Tissue sample	Cell Line and Liquid Cancer samples	Especially for needle biopsy solid samples
Homogenization method for tissue sample	Not explored	Tissue sample homogenized using a liquid nitrogen-cooled mortar and pestle	Not explored	Tissue sample homogenized using a liquid nitrogen-cooled mortar and pestle
End filling	Biotin-dependent strategy	Biotin-free strategy	Biotin-dependent strategy	Biotin-dependent strategy
Method	The cross linked chromatin is digested with a restriction	Involves a series of enzymatic reactions.	The cross linked chromatin is digested with a restriction	The cross linked chromatin is digested with a restriction

	enzyme. The ends are filled in with biotinylated nucleotides followed by ligation. After ligation, crosslinks were reversed, and the DNA was purified from protein. The purified proximity ligated DNA was sheared and size-selected from ~200-600bp. The size-selected fragments were then enriched for biotin and converted into Illumina-compatible sequencing libraries.	The crosslinked chromatin is digested with a restriction enzyme followed by *in situ* proximity ligation without end repair. After reverse crosslinking, the DNA is digested with more frequent 4-base cutter *DpnII* before self-ligation. Exonuclease was used to remove DNA that failed to form circles, as well as contaminations from un-ligated ends and other linear DNA species. The circularized DNA was again cut with HindIII. The re-linearized junction DNA was only sequenced.	enzyme. The ends are filled in with biotinylated nucleotides followed by ligation. After ligation, crosslinks were reversed, and the DNA was purified from protein. The purified proximity ligated DNA was sheared and size-selected from ~200-600bp. The size-selected fragments were then enriched for biotin and converted into Illumina-compatible sequencing libraries.	enzyme cocktail. The resulting overhangs were filled in with biotinylated nucleotides, followed by ligation. After ligation, crosslinks were reversed, and the DNA was purified from protein. The purified proximity ligated DNA was sheared and size-selected from ~200-600bp. The size-selected fragments were then enriched for biotin and converted into Illumina-compatible sequencing libraries.
Starting Material	500 cells	50–100k cells	Up to 1000 cells	Needle Biopsy Sample

Table S2

	**S009**	**S010**	**S012**	**S024**
Gender	Male	Male	Male	Unknown
Age	55	58	48	Unknown
Biopsy site	Liver	Pelvic	PNS	Unknown
Needle size	18G	16G/18G	-	Unknown

PNS: posterior nasal space

Supplementary Figures

Fig S1.

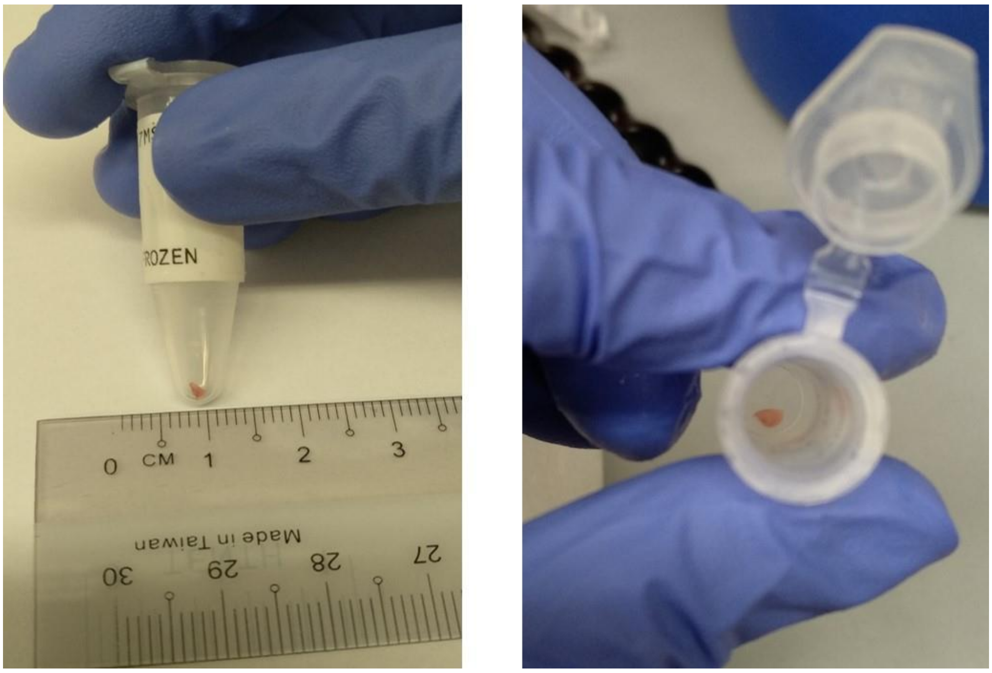

Fig S2A

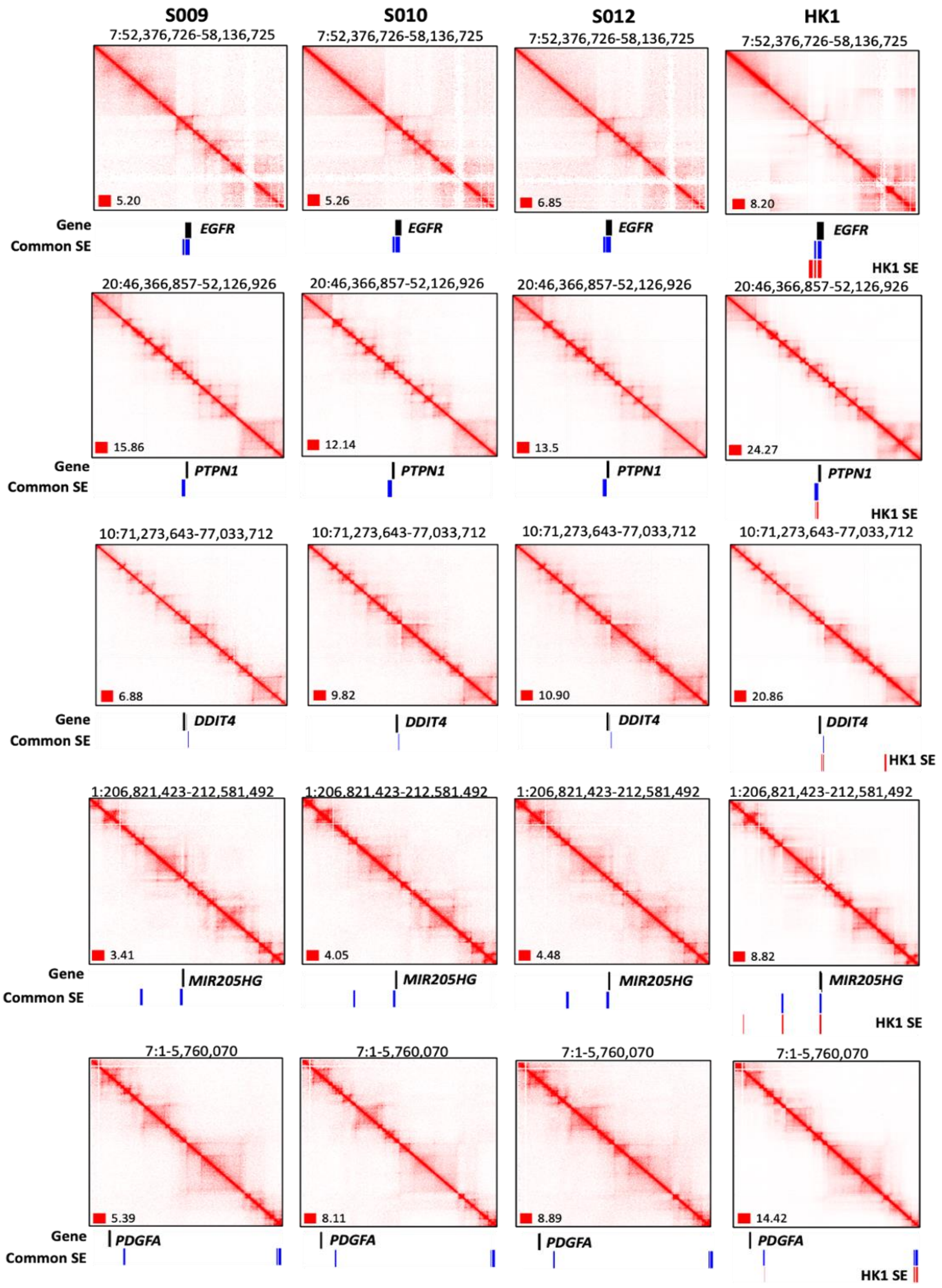
S009
S010
S012
HK1
7:52,376,726-58,136,725
5.20
5.26
6.85
8.20
Gene
Common SE
EGFR
HK1 SE
20:46,366,857-52,126,926
15.86
12.14
13.5
24.27
PTPN1
10:71,273,643-77,033,712
6.88
9.82
10.90
20.86
DDIT4
1:206,821,423-212,581,492
3.41
4.05
4.48
8.82
MIR205HG
7:1-5,760,070
5.39
8.11
8.89
14.42
PDGFA

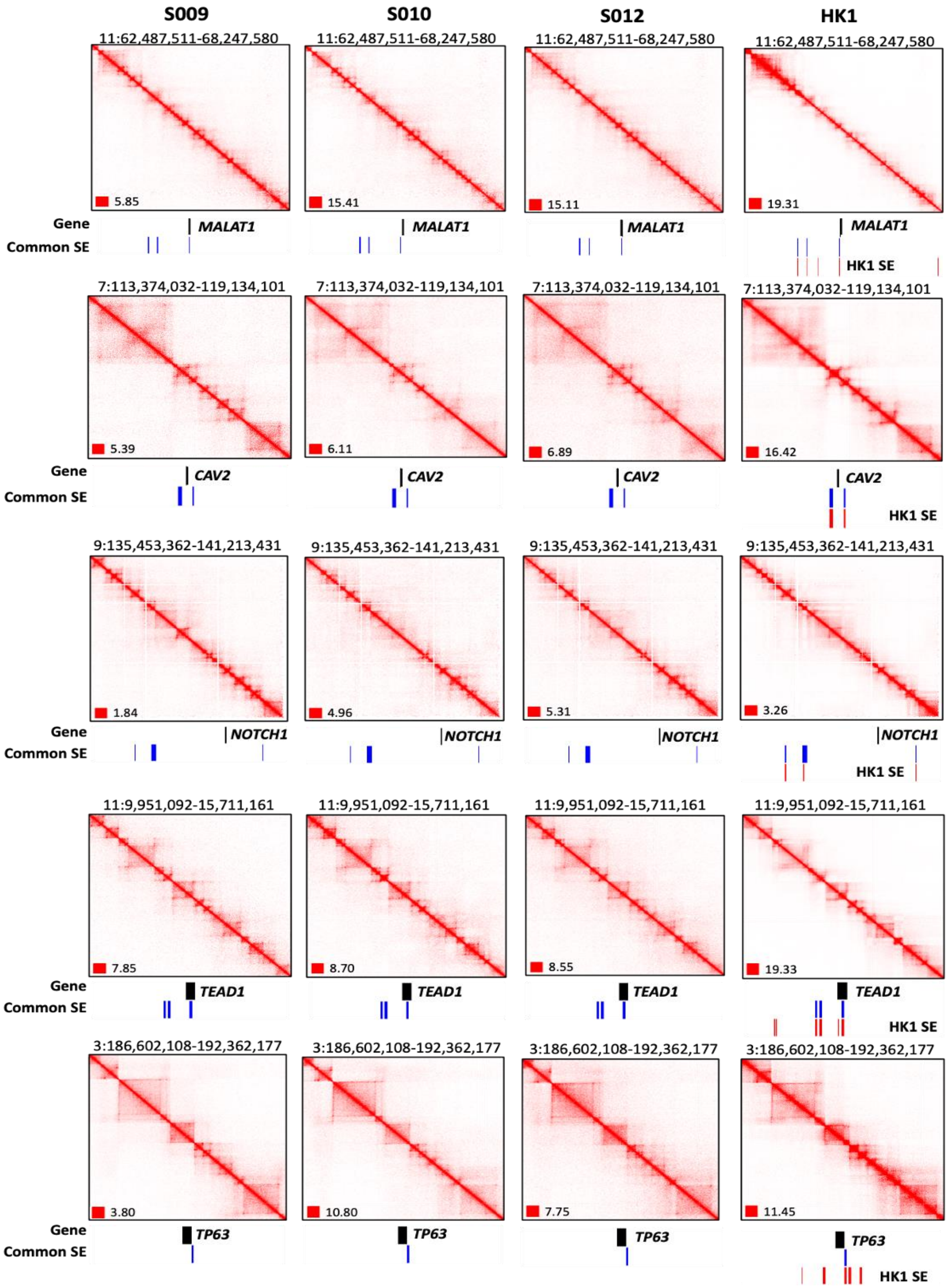

S009
S010
S012
HK1
11:62,487,511-68,247,580
5.85
15.41
15.11
19.31
Gene
Common SE
MALAT1
HK1 SE
7:113,374,032-119,134,101
5.39
6.11
6.89
16.42
CAV2
9:135,453,362-141,213,431
1.84
4.96
5.31
3.26
NOTCH1
11:9,951,092-15,711,161
7.85
8.70
8.55
19.33
TEAD1
3:186,602,108-192,362,177
3.80
10.80
7.75
11.45
TP63

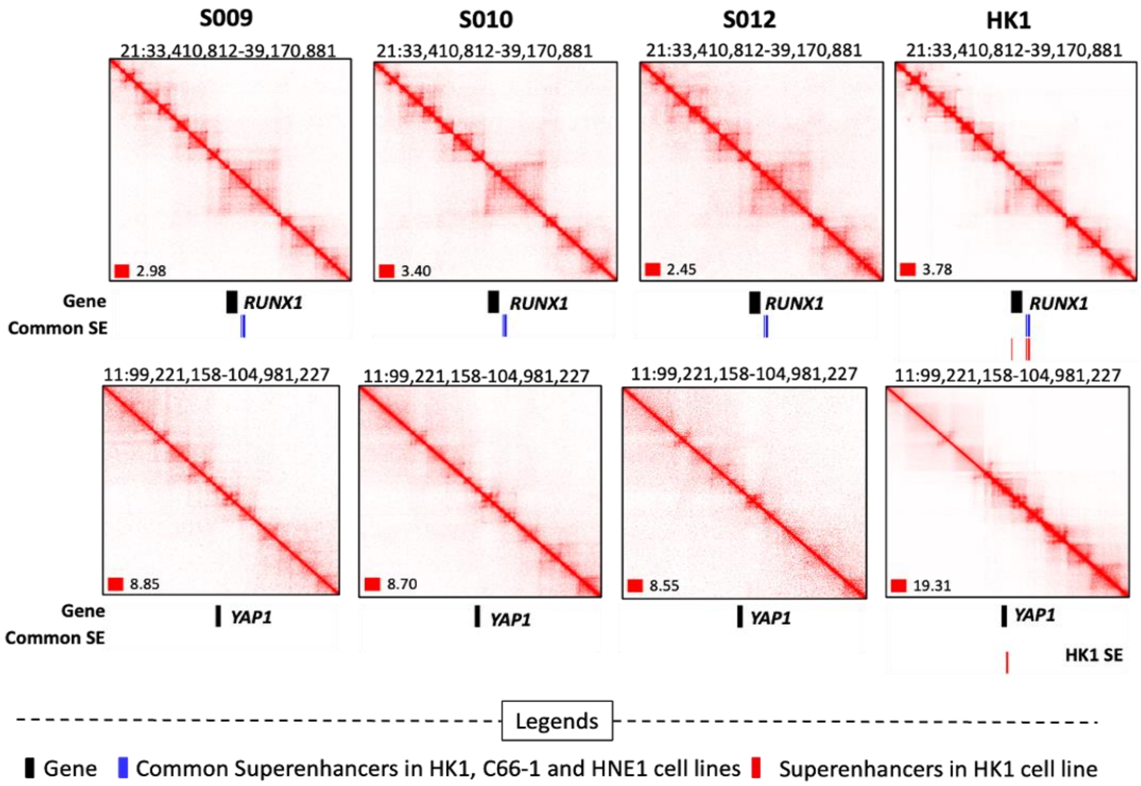

S009
S010
S012
HK1
21:33,410,812-39,170,881
2.98
3.40
2.45
3.78
Gene
Common SE
RUNX1
11:99,221,158-104,981,227
8.85
8.70
8.55
19.31
YAP1
HK1 SE
Legends
Gene
Common Superenhancers in HK1, C66-1 and HNE1 cell lines
Superenhancers in HK1 cell line

Fig S2B

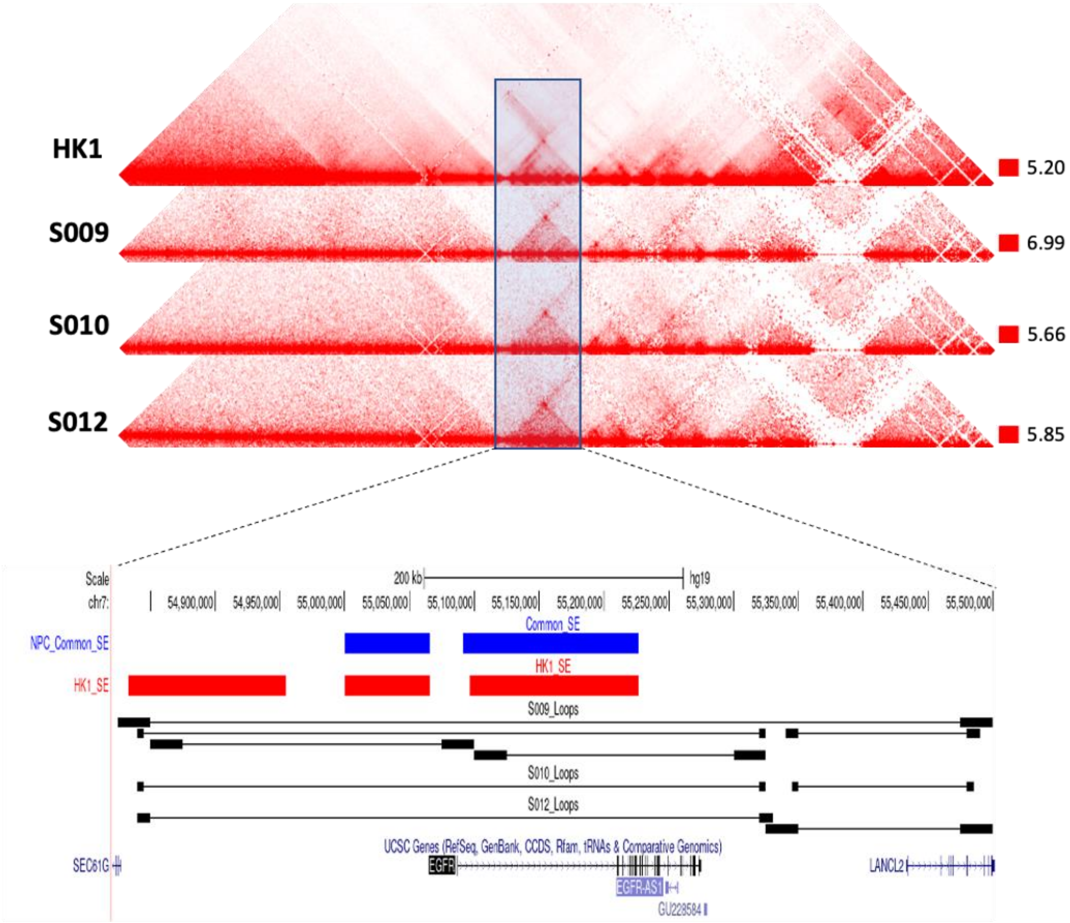

Fig S2C

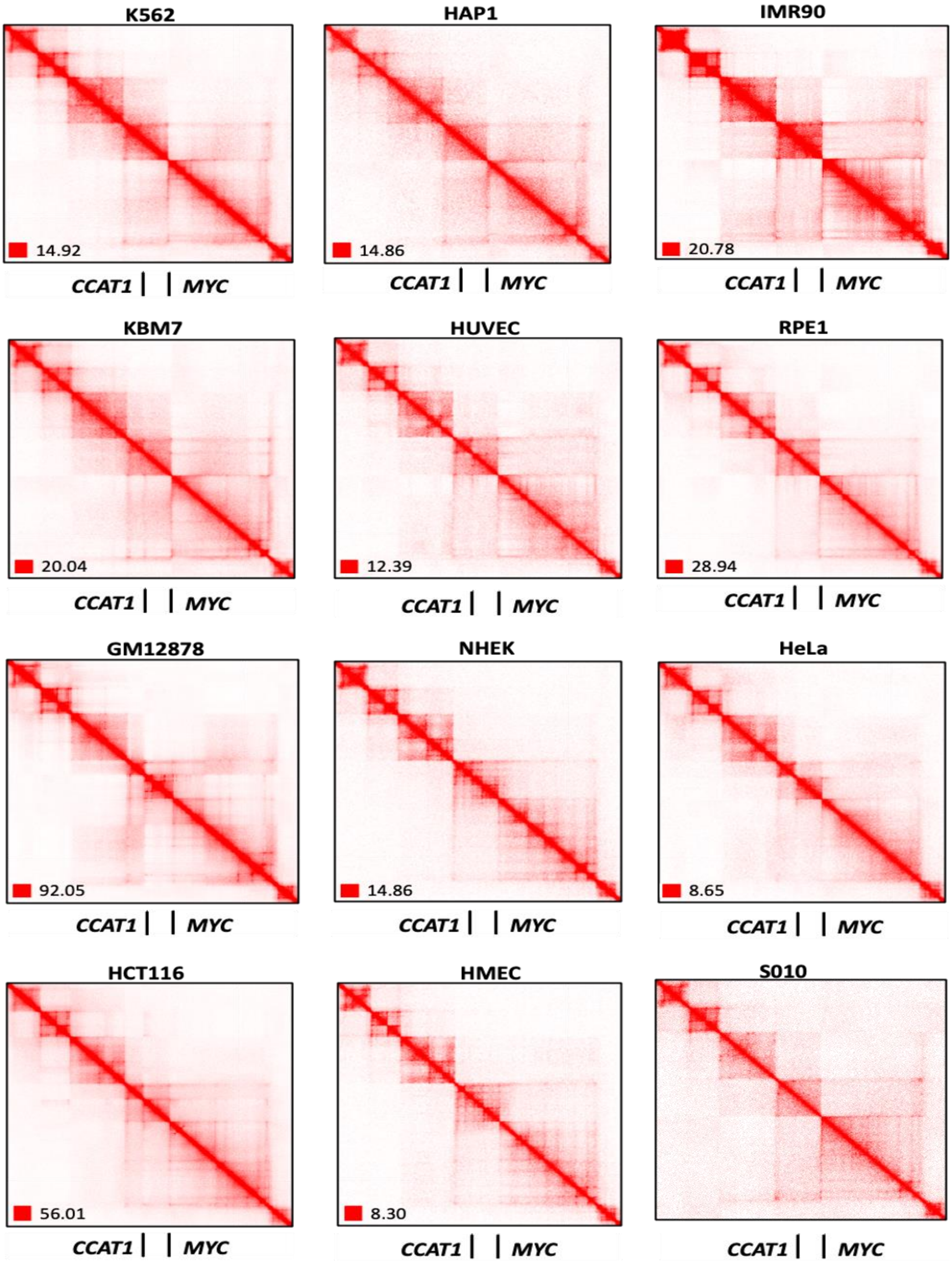
K562
14.92
CCAT1
MYC
HAP1
14.86
CCAT1
MYC
IMR90
20.78
CCAT1
MYC
KBM7
20.04
CCAT1
MYC
HUVEC
12.39
CCAT1
MYC
RPE1
28.94
CCAT1
MYC
GM12878
92.05
CCAT1
MYC
NHEK
14.86
CCAT1
MYC
HeLa
8.65
CCAT1
MYC
HCT116
56.01
CCAT1
MYC
HMEC
8.30
CCAT1
MYC
S010
CCAT1
MYC

Fig S2D

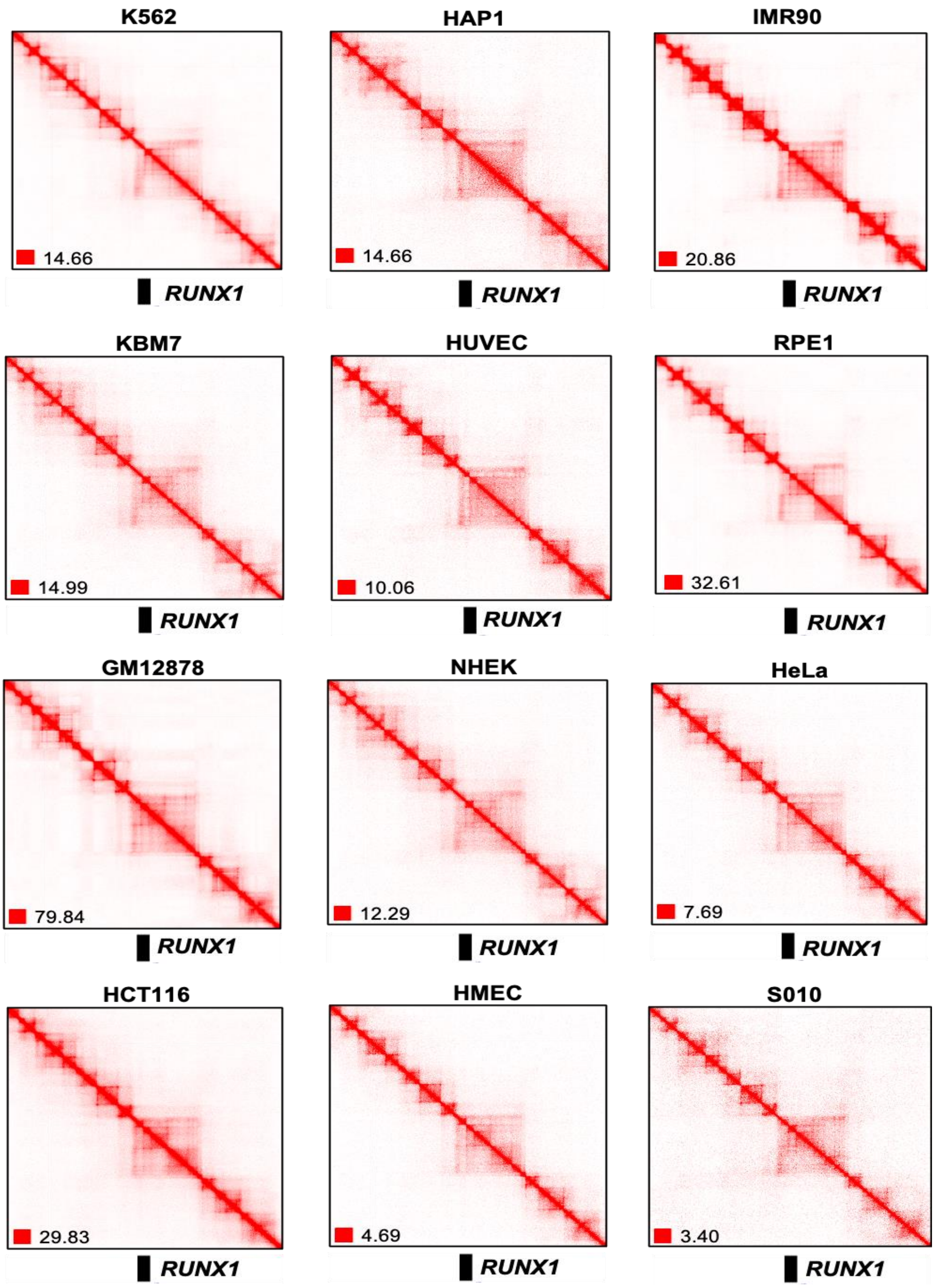
K562
14.66
RUNX1
HAP1
14.66
RUNX1
IMR90
20.86
RUNX1
KBM7
14.99
RUNX1
HUVEC
10.06
RUNX1
RPE1
32.61
RUNX1
GM12878
79.84
RUNX1
NHEK
12.29
RUNX1
HeLa
7.69
RUNX1
HCT116
29.83
RUNX1
HMEC
4.69
RUNX1
S010
3.40
RUNX1

Fig S2E

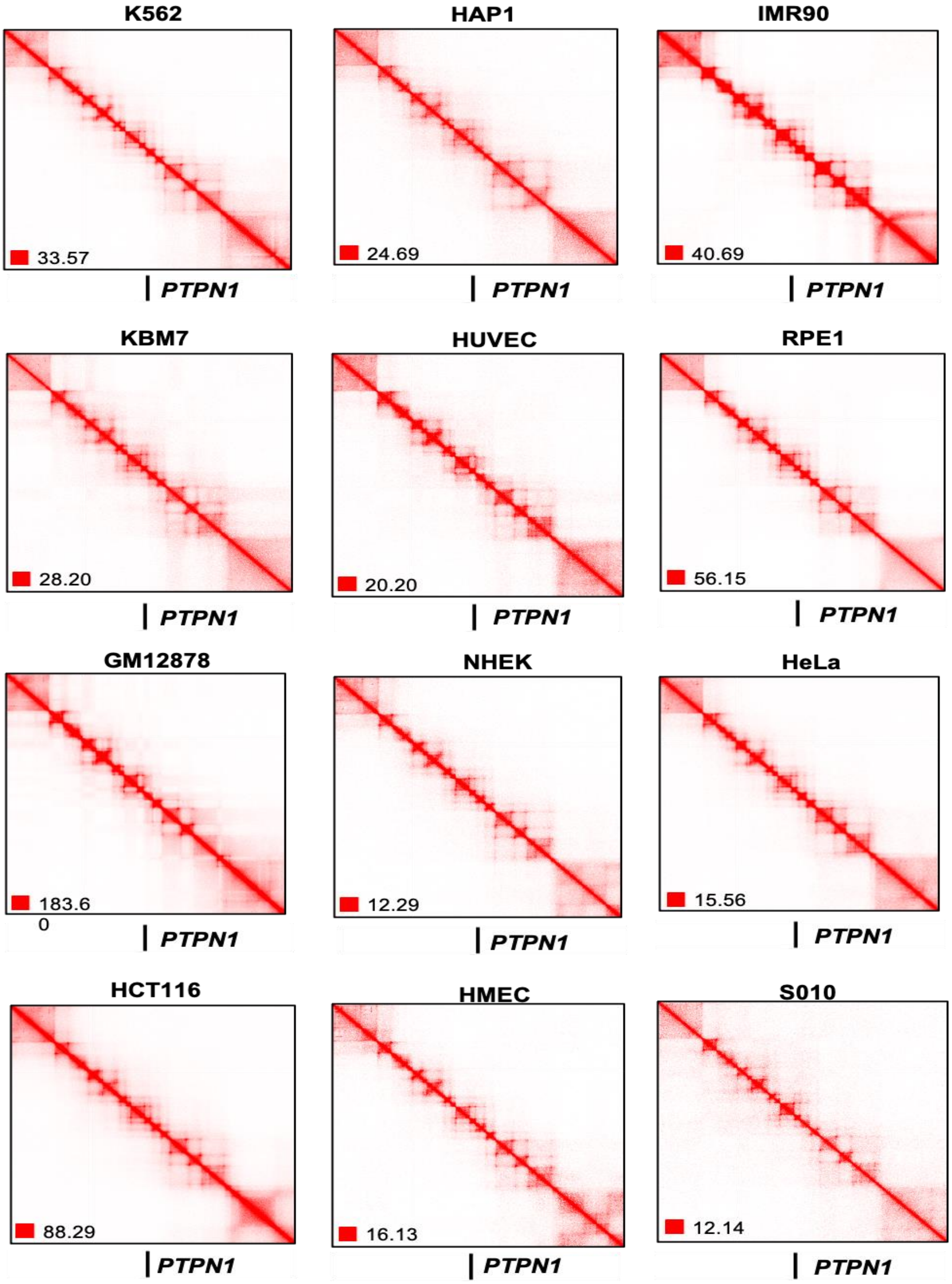
K562
33.57
PTPN1
HAP1
24.69
PTPN1
IMR90
40.69
PTPN1
KBM7
28.20
PTPN1
HUVEC
20.20
PTPN1
RPE1
56.15
PTPN1
GM12878
183.6
0
PTPN1
NHEK
12.29
PTPN1
HeLa
15.56
PTPN1
HCT116
88.29
PTPN1
HMEC
16.13
PTPN1
S010
12.14
PTPN1

Fig S2F

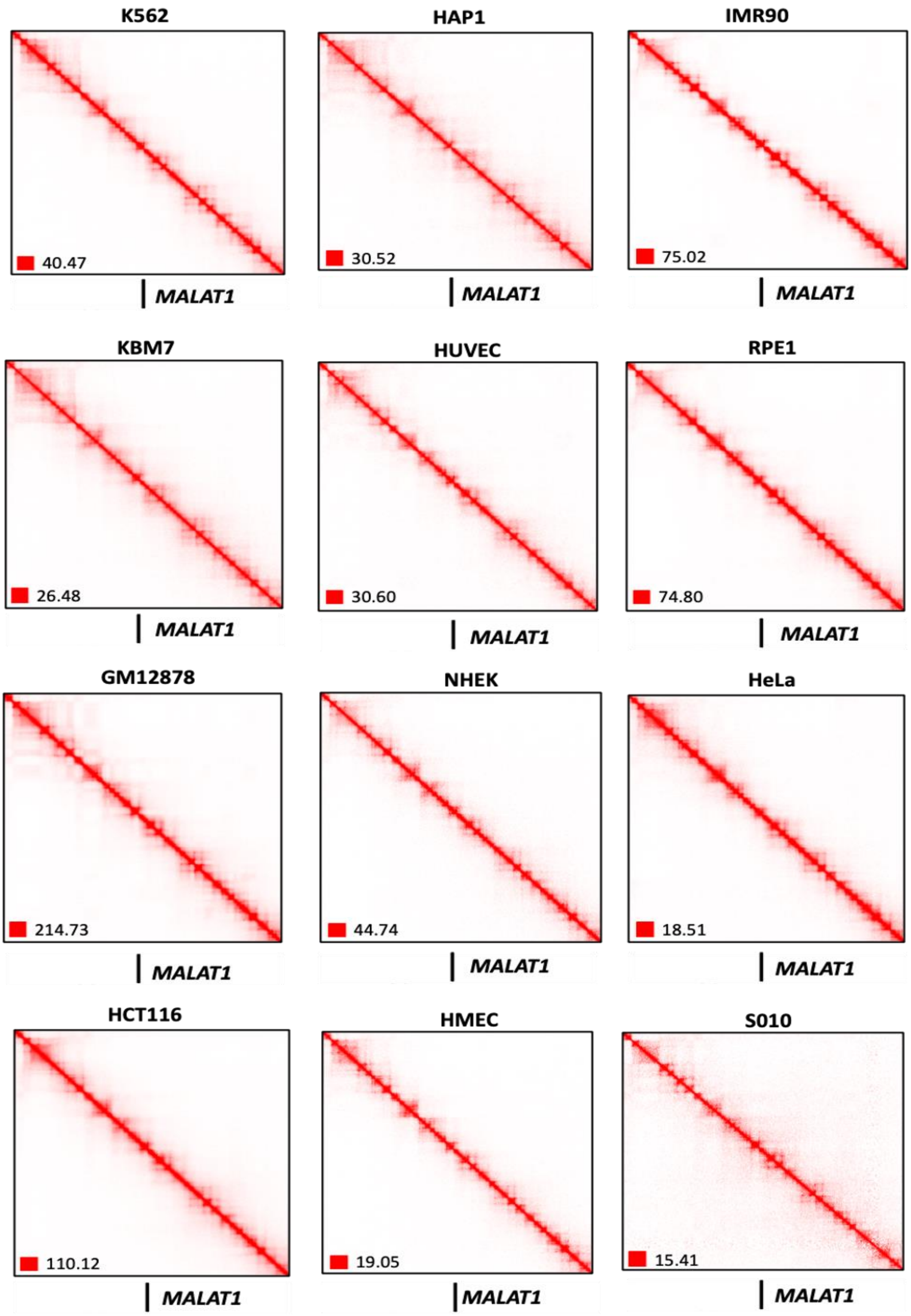
K562
40.47
MALAT1
HAP1
30.52
MALAT1
IMR90
75.02
MALAT1
KBM7
26.48
MALAT1
HUVEC
30.60
MALAT1
RPE1
74.80
MALAT1
GM12878
214.73
MALAT1
NHEK
44.74
MALAT1
HeLa
18.51
MALAT1
HCT116
110.12
MALAT1
HMEC
19.05
MALAT1
S010
15.41
MALAT1

Fig S3A

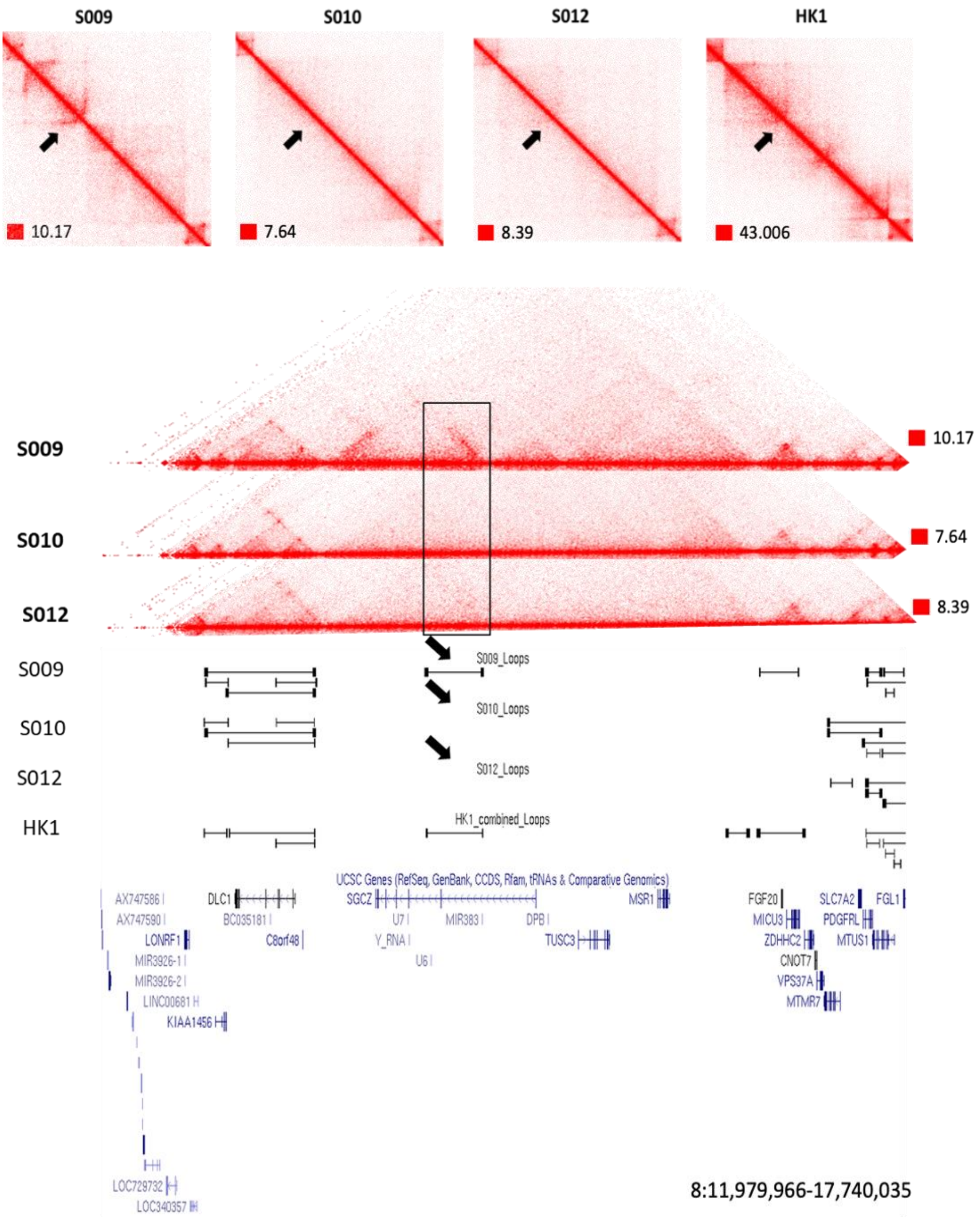
S009
S010
S012
HK1
10.17
7.64
8.39
43.006
S009
S010
S012
10.17
7.64
8.39
S009
S010
S012
HK1
S009_Loops
S010_Loops
S012_Loops
HK1_combined_Loops
UCSC Genes (RefSeq, GenBank, CCDS, Rfam, tRNAs & Comparative Genomics)
AX747586
AX747590
LONRF1
MIR3926-1
MIR3926-2
LINC00681
KIAA1456
LOC729732
LOC340357
DLC1
BC035181
C8orf48
SGCZ
U7
Y_RNA
U6
MIR383
DPB
TUSC3
MSR1
FGF20
MICU3
ZDHHC2
CNOT7
VPS37A
MTMR7
SLC7A2
PDGFRL
MTUS1
FGL1
8:11,979,966-17,740,035

Fig S3B

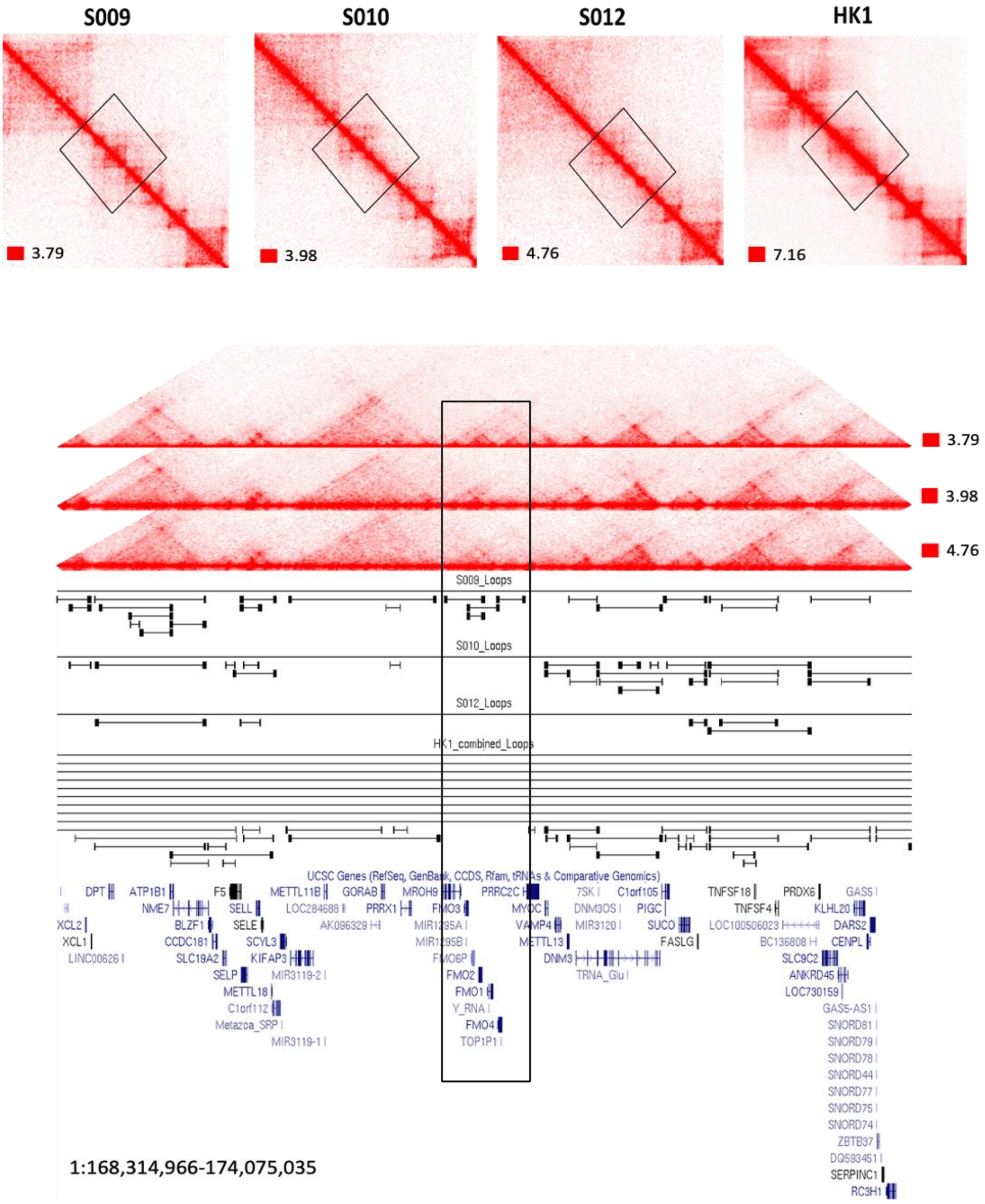
S009
S010
S012
HK1
3.79
3.98
4.76
7.16
3.79
3.98
4.76
S009_Loops
S010_Loops
S012_Loops
HK1_combined_Loops
UCSC Genes (RefSeq, GenBank, CCDS, Rfam, tRNAs & Comparative Genomics)
DPT
ATP1B1
F5
METTL11B
GORAB
MROH9
PRRC2C
7SK
C1orf105
TNFSF18
PRDX6
GAS5
NME7
SELL
LOC284688
PRRX1
FMO3
MYOC
DNM3OS
PIGC
TNFSF4
KLHL20
XCL2
BLZF1
SELE
AK096329
MIR1295A
VAMP4
MIR3120
SUCO
LOC100506023
DARS2
XCL1
CCDC181
SCYL3
MIR1295B
METTL13
FASLG
BC136808
CENPL
LINC00626
SLC19A2
KIFAP3
FMO6P
DNM3
SLC9C2
SELP
MIR3119-2
FMO2
TRNA_Glu
ANKRD45
METTL18
FMO1
LOC730159
C1orf112
Y_RNA
GAS5-AS1
Metazoa_SRP
FMO4
SNORD81
MIR3119-1
TOP1P1
SNORD79
SNORD78
SNORD44
SNORD77
SNORD75
SNORD74
ZBTB37
DQ593451
SERPINC1
RC3H1
1:168,314,966-174,075,035

Fig S4

A

Sample ID	# HiC-contacts	# TADs	# Loops	# FIREs
NP-69	523,683,401	5227	11415	2893

B

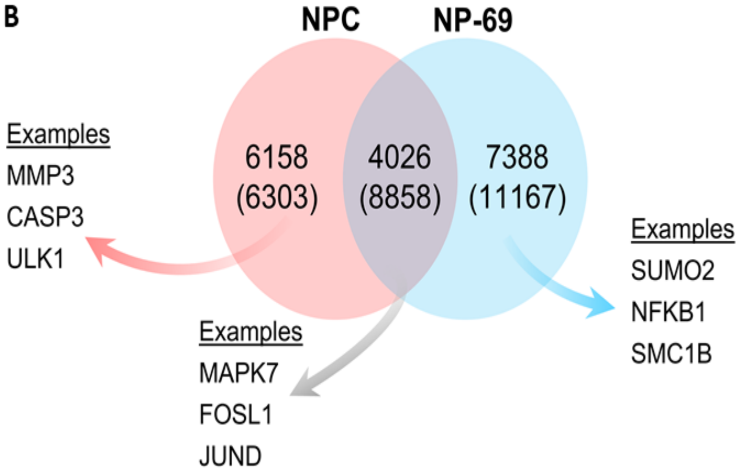

Fig S5 A-B

A

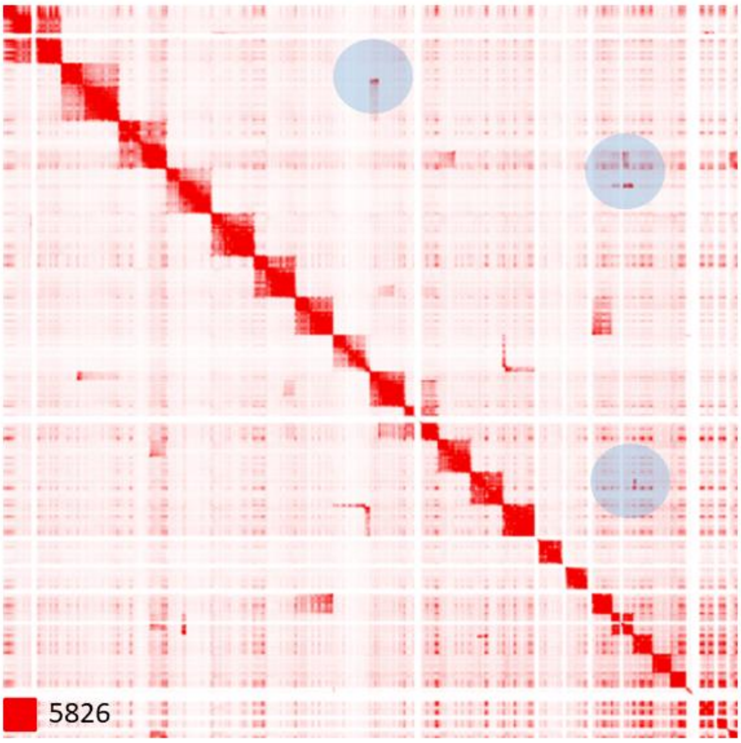

S009

B

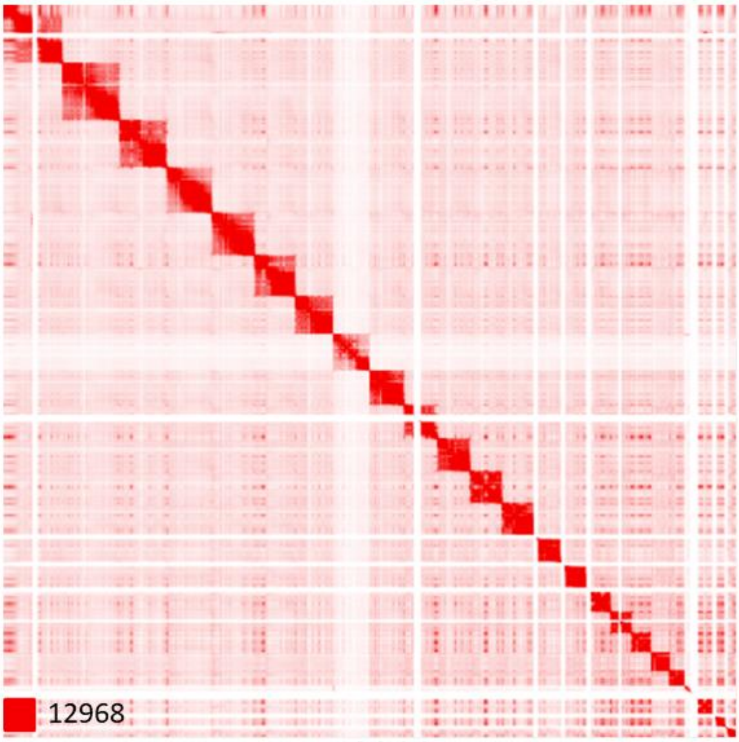

S010

Fig S5 C-D

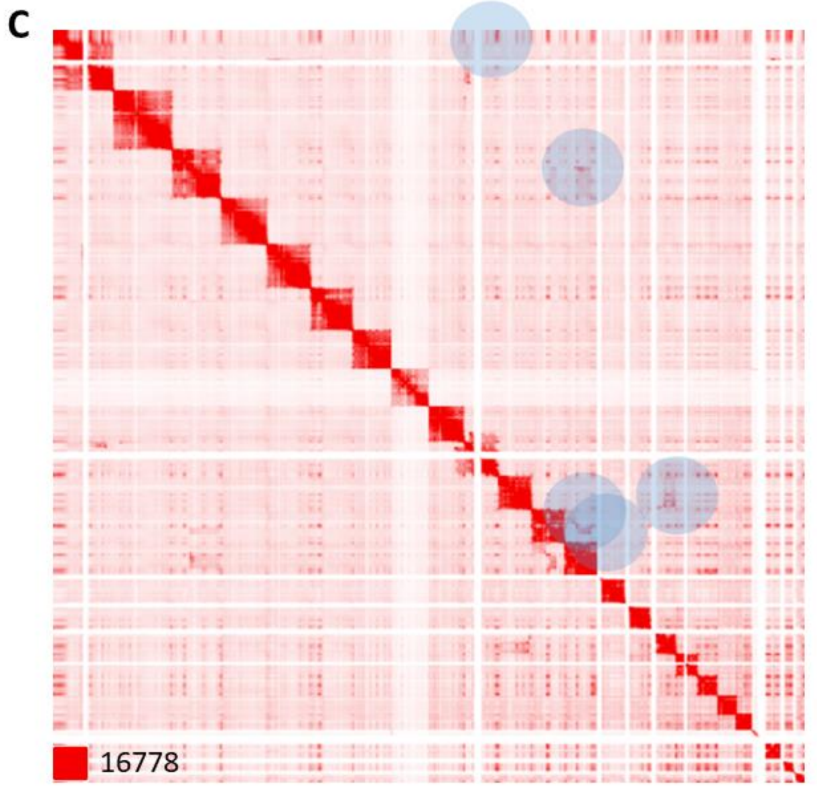

S012

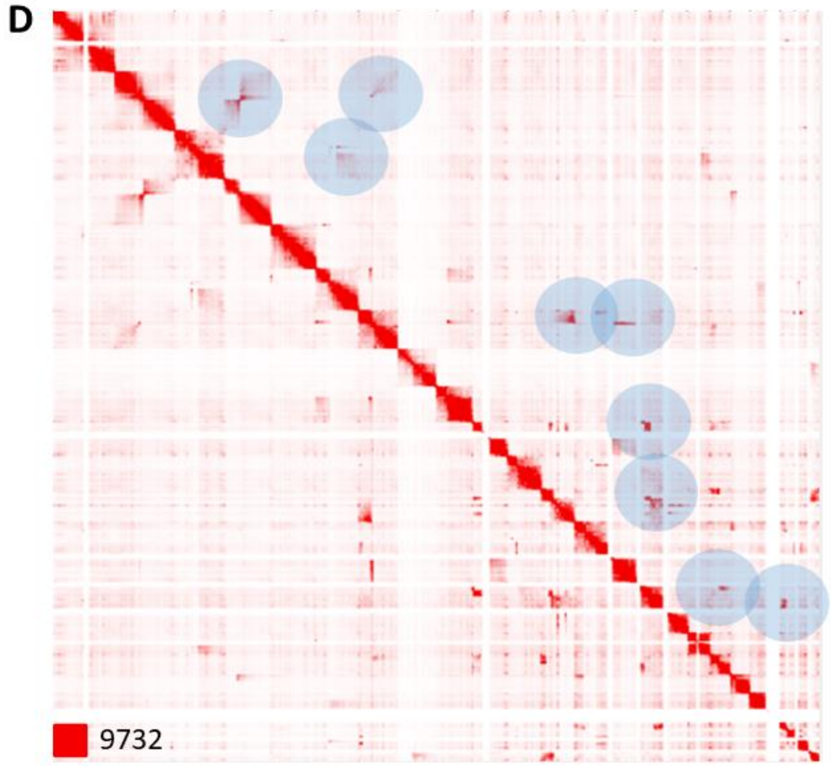

HK1

Fig S5 E

E

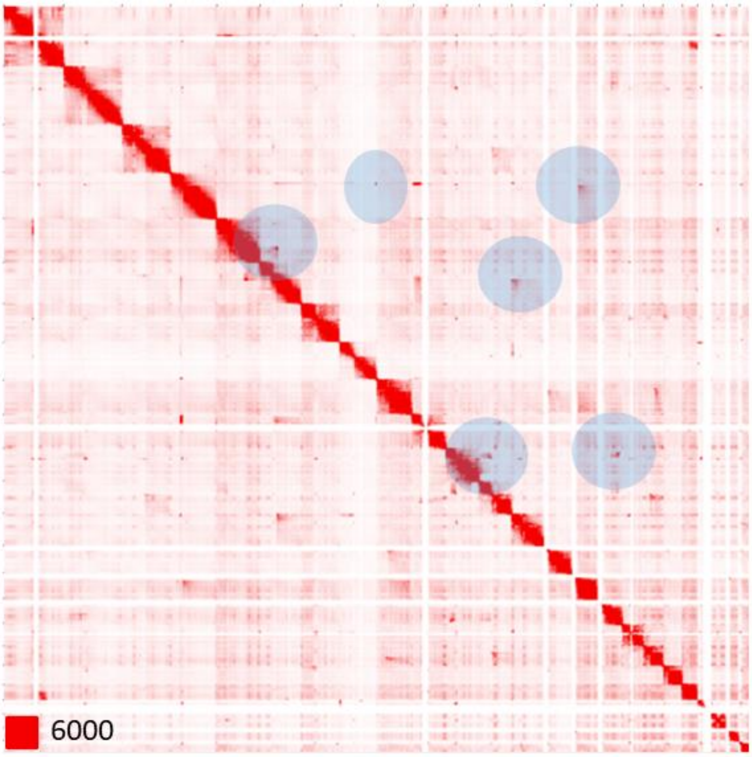

NP69

Fig S6

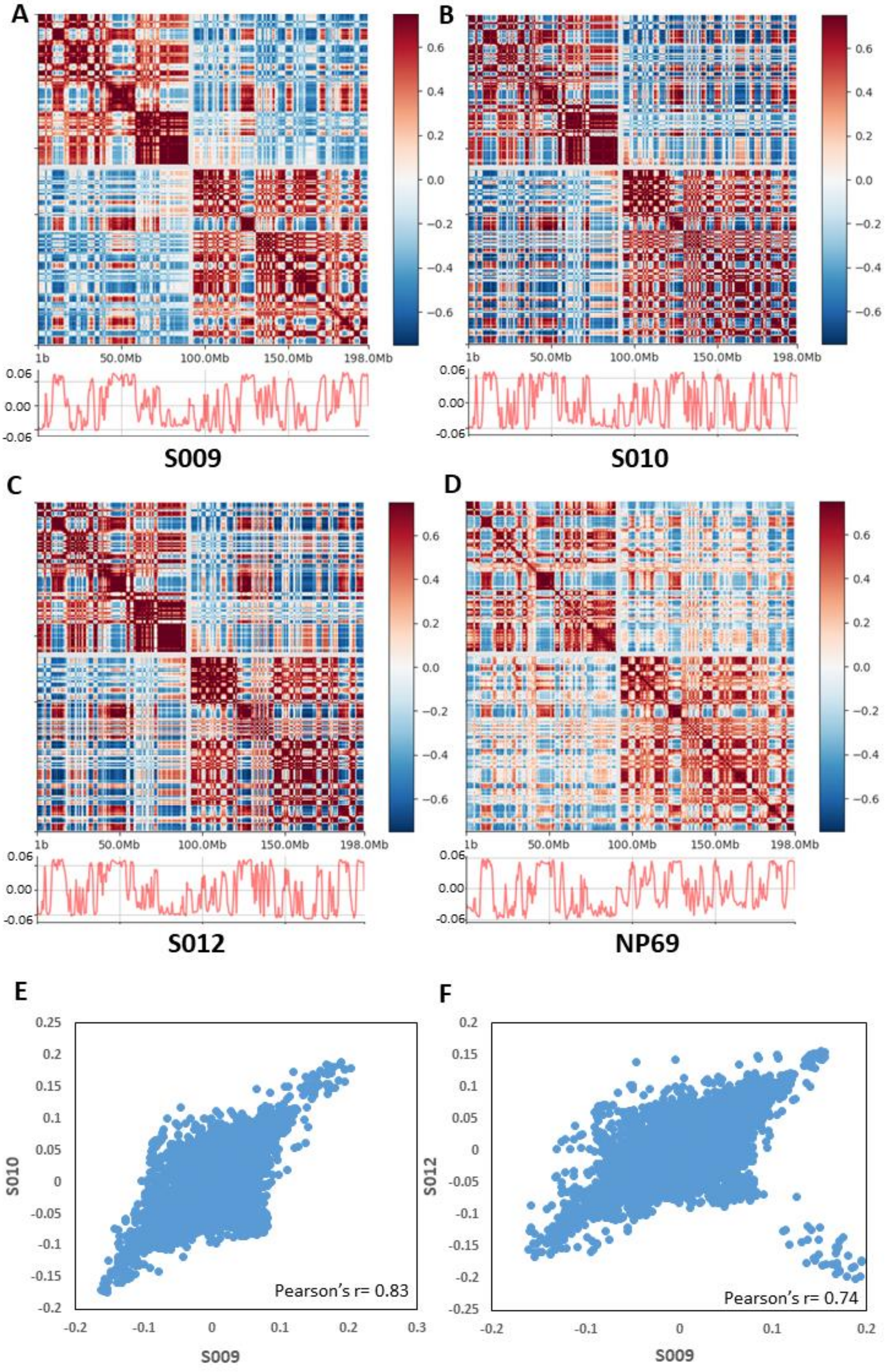
A
B
C
D
S009
S010
S012
NP69
1b
50.0Mb
100.0Mb
150.0Mb
198.0Mb
E
F
Pearson's r= 0.83
Pearson's r= 0.74
S010
S012
S009

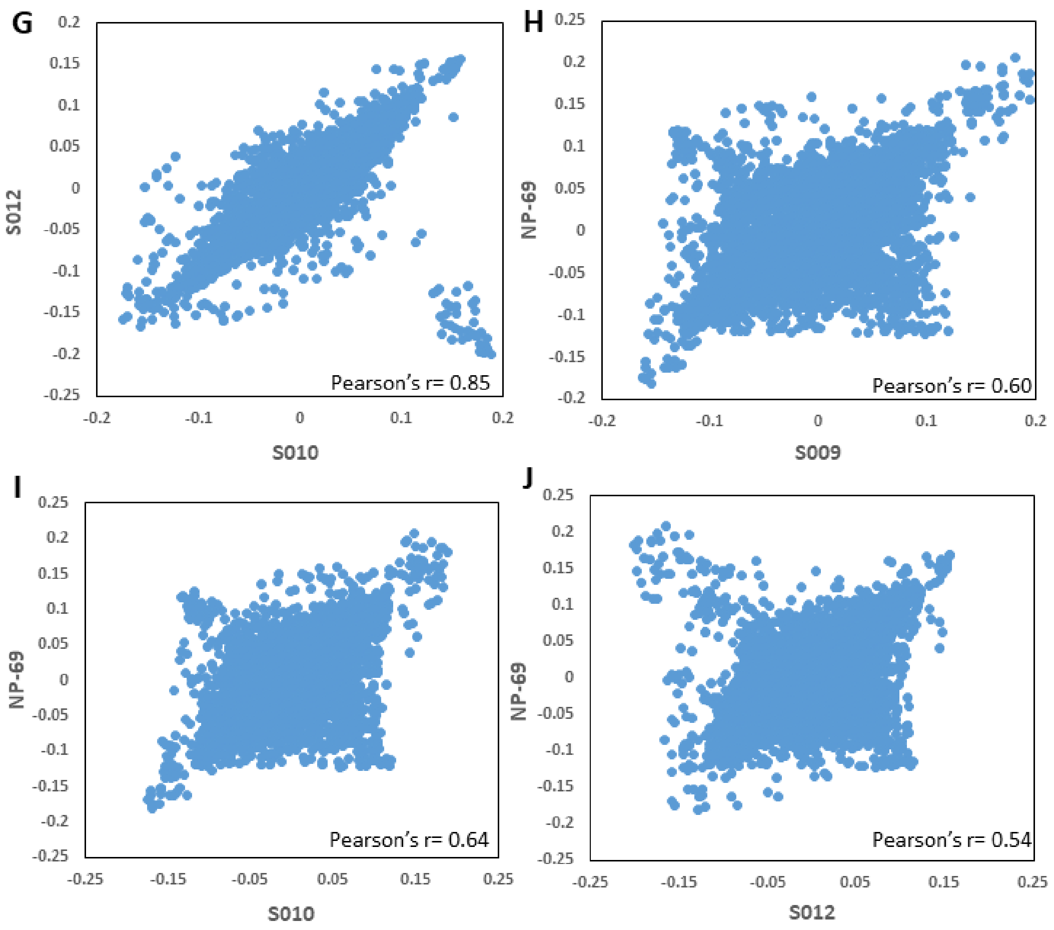
G
S012
S010
Pearson's r= 0.85
H
NP-69
S009
Pearson's r= 0.60
I
NP-69
S010
Pearson's r= 0.64
J
NP-69
S012
Pearson's r= 0.54

Fig S7

A

Sample ID	# HiC-contacts	# TADs	# Loops	# FIREs
S024_R1	278,588,162	247	5094	1707
S024_R2	261,921,134	8	2199	2893

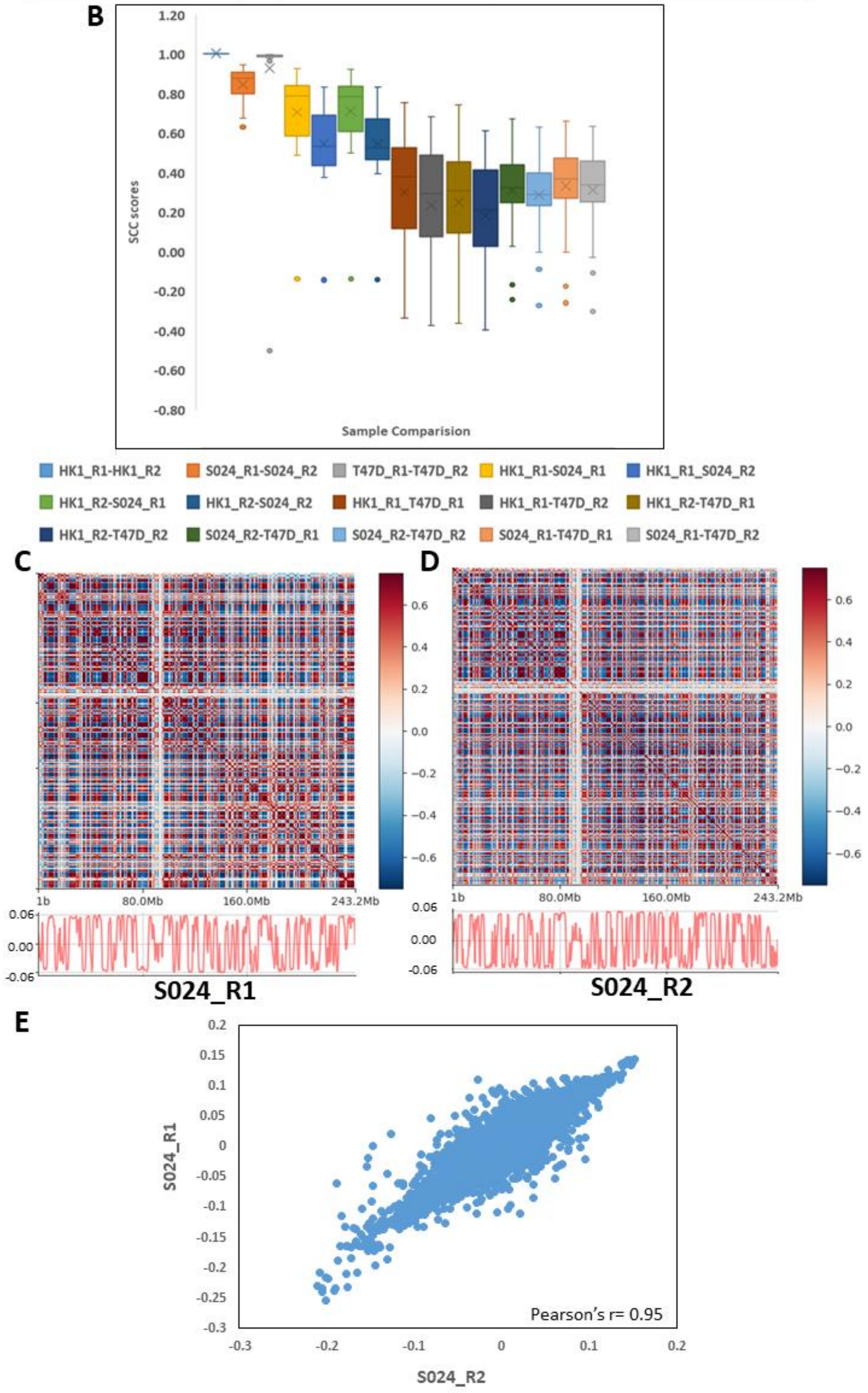

Fig S8

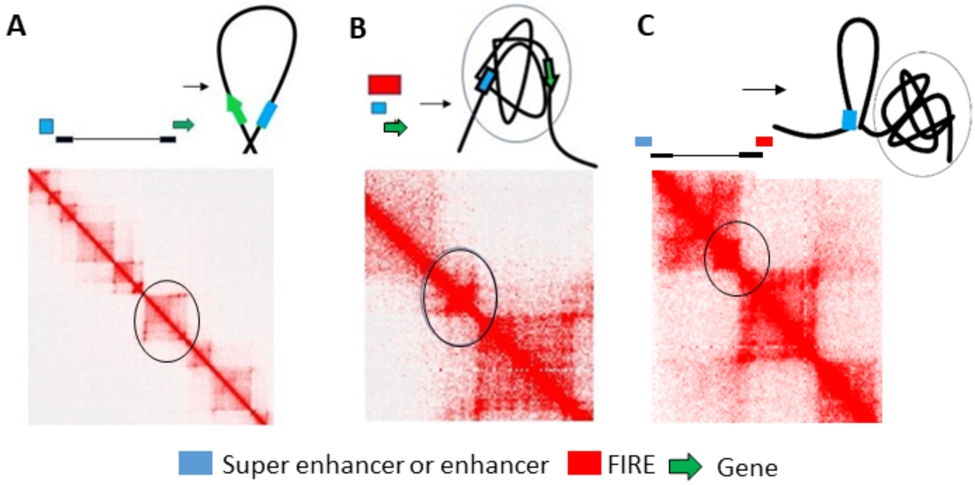
A
B
C
Super enhancer or enhancer
FIRE
Gene

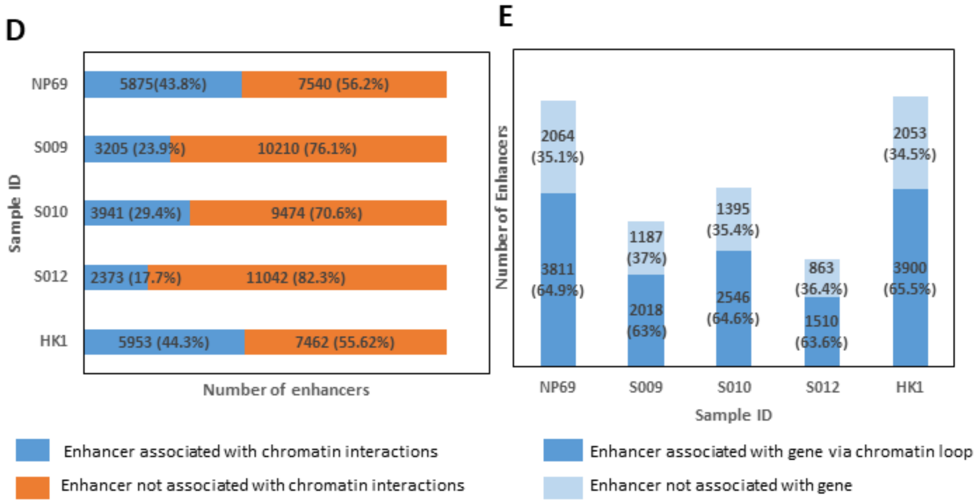
D
NP69
S009
S010
S012
HK1
Sample ID
5875(43.8%)
7540 (56.2%)
3205 (23.9%)
10210 (76.1%)
3941 (29.4%)
9474 (70.6%)
2373 (17.7%)
11042 (82.3%)
5953 (44.3%)
7462 (55.62%)
Number of enhancers
Enhancer associated with chromatin interactions
Enhancer not associated with chromatin interactions
E
Number of Enhancers
2064 (35.1%)
3811 (64.9%)
1187 (37%)
2018 (63%)
1395 (35.4%)
2546 (64.6%)
863 (36.4%)
1510 (63.6%)
2053 (34.5%)
3900 (65.5%)
NP69
S009
S010
S012
HK1
Sample ID
Enhancer associated with gene via chromatin loop
Enhancer not associated with gene

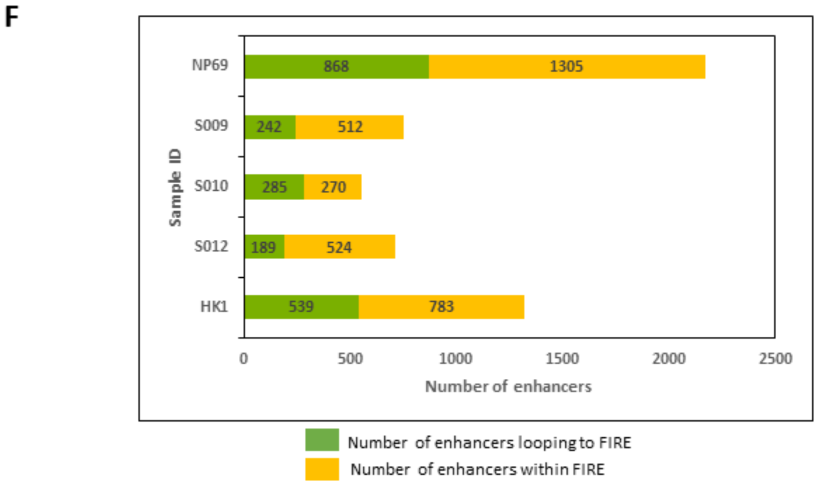
F
NP69
S009
S010
S012
HK1
Sample ID
868
1305
242
512
285
270
189
524
539
783
0
500
1000
1500
2000
2500
Number of enhancers
Number of enhancers looping to FIRE
Number of enhancers within FIRE

Fig S9

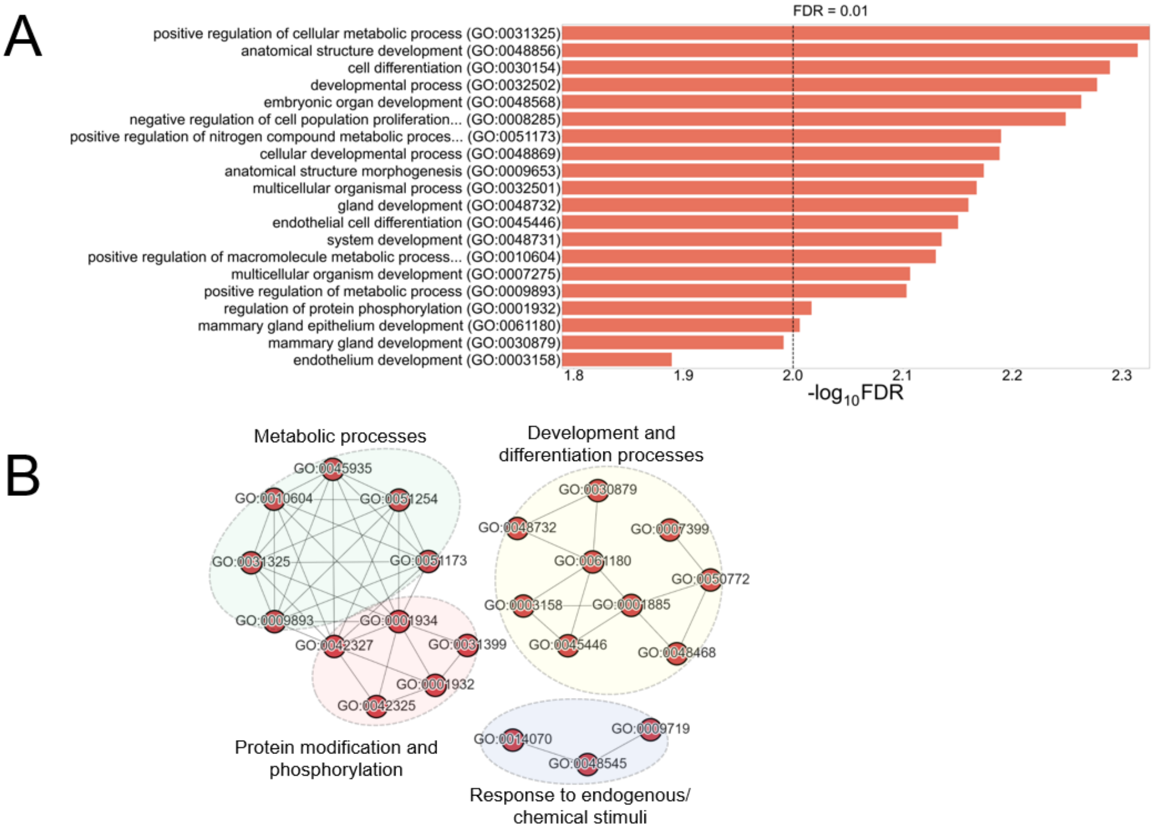
A
FDR = 0.01
positive regulation of cellular metabolic process (GO:0031325)
anatomical structure development (GO:0048856)
cell differentiation (GO:0030154)
developmental process (GO:0032502)
embryonic organ development (GO:0048568)
negative regulation of cell population proliferation... (GO:0008285)
positive regulation of nitrogen compound metabolic proces... (GO:0051173)
cellular developmental process (GO:0048869)
anatomical structure morphogenesis (GO:0009653)
multicellular organismal process (GO:0032501)
gland development (GO:0048732)
endothelial cell differentiation (GO:0045446)
system development (GO:0048731)
positive regulation of macromolecule metabolic process... (GO:0010604)
multicellular organism development (GO:0007275)
positive regulation of metabolic process (GO:0009893)
regulation of protein phosphorylation (GO:0001932)
mammary gland epithelium development (GO:0061180)
mammary gland development (GO:0030879)
endothelium development (GO:0003158)
1.8
1.9
2.0
2.1
2.2
2.3
$-\log_{10}$FDR
B
Metabolic processes
GO:0045935
GO:0010604
GO:0051254
GO:0031325
GO:0051173
GO:0009893
GO:0001934
GO:0042327
GO:0031399
GO:0001932
GO:0042325
Protein modification and phosphorylation
Development and differentiation processes
GO:0030879
GO:0048732
GO:0007399
GO:0061180
GO:0050772
GO:0003158
GO:0001885
GO:0045446
GO:0048468
GO:0014070
GO:0009719
GO:0048545
Response to endogenous/ chemical stimuli

Fig S10

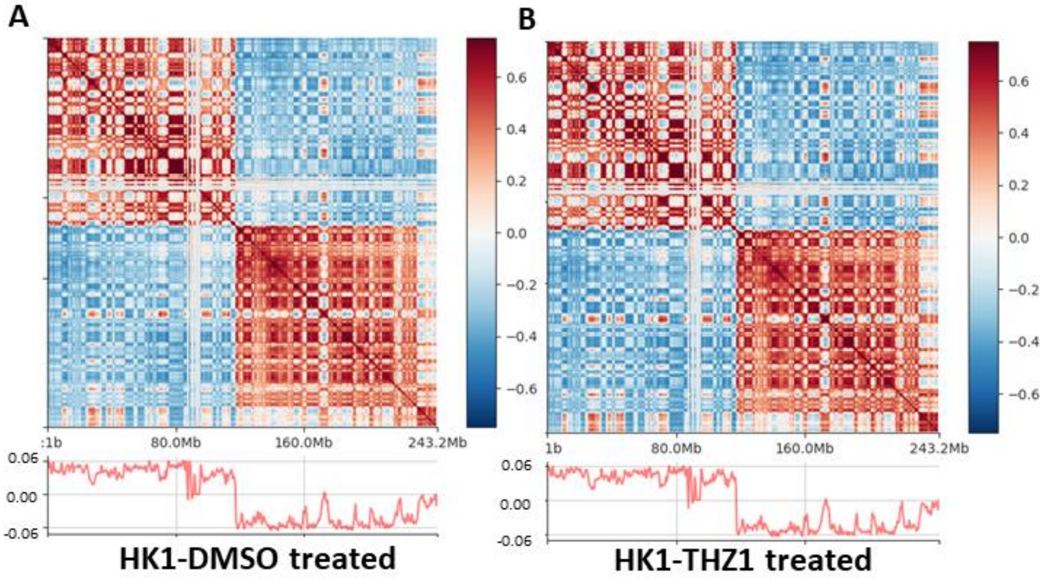
A
B
0.6
0.4
0.2
0.0
−0.2
−0.4
−0.6
:1b
80.0Mb
160.0Mb
243.2Mb
1b
0.06
0.00
-0.06
HK1-DMSO treated
HK1-THZ1 treated

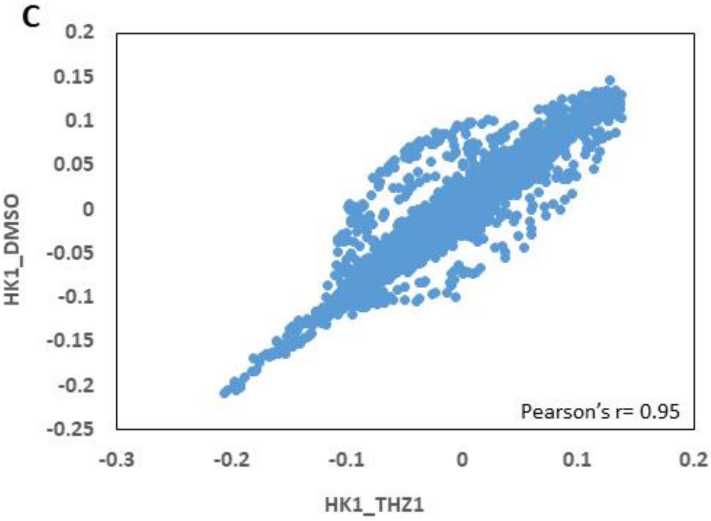
C
0.2
0.15
0.1
0.05
0
-0.05
-0.1
-0.15
-0.2
-0.25
HK1_DMSO
-0.3
-0.2
-0.1
0
0.1
0.2
HK1_THZ1
Pearson's r= 0.95

Supplementary References

Darrow, E. M., Huntley, M. H., Dudchenko, O., Stamenova, E. K., Durand, N. C., Sun, Z., et al. (2016). Deletion of DXZ4 on the human inactive X chromosome alters higher-order genome architecture. *Proc. Natl. Acad. Sci. U. S. A.* 113, E4504–E4512. doi:10.1073/pnas.1609643113.

Díaz, N., Kruse, K., Erdmann, T., Staiger, A. M., Ott, G., Lenz, G., et al. (2018). Chromatin conformation analysis of primary patient tissue using a low input Hi-C method. *Nat. Commun.* 9, 1–13. doi:10.1038/s41467-018-06961-0.

Du, Z., Zheng, H., Huang, B., Ma, R., Wu, J., Zhang, X., et al. (2017). Allelic reprogramming of 3D chromatin architecture during early mammalian development. *Nature* 547, 232–235. doi:10.1038/nature23263.

Haarhuis, J. H. I., van der Weide, R. H., Blomen, V. A., Yáñez-Cuna, J. O., Amendola, M., van Ruiten, M. S., et al. (2017). The Cohesin Release Factor WAPL Restricts Chromatin Loop Extension. *Cell* 169, 693-707.e14. doi:10.1016/j.cell.2017.04.013.

Ke, L., Zhou, H., Wang, C., Xiong, G., Xiang, Y., Ling, Y., et al. (2017). Super-enhancers promote transcriptional dysregulation in nasopharyngeal carcinoma. *Proc. Natl. Acad. Sci. U. S. A.* 77, 6614–6626. doi:10.1158/0008-5472.CAN-17-1143.

Lu, L., Liu, X., Huang, W. K., Giusti-Rodríguez, P., Cui, J., Zhang, S., et al. (2020). Robust Hi-C Maps of Enhancer-Promoter Interactions Reveal the Function of Non-coding Genome in Neural Development and Diseases. *Mol. Cell* 79, 521-534.e15. doi:10.1016/J.MOLCEL.2020.06.007.

Rao, S. S. P., Huang, S.-C., Glenn, B., Hilaire, S., Casellas, R., Lander, E. S., et al. (2017). Cohesin Loss Eliminates All Loop Domains. *Cell* 171, 305–320. doi:10.1016/j.cell.2017.09.026.

Rao, S. S. P., Huntley, M. H., Durand, N. C., Stamenova, E. K., Bochkov, I. D., Robinson, J. T., et al. (2014). A 3D map of the human genome at kilobase resolution reveals principles of chromatin looping. *Cell* 159, 1665–1680. doi:10.1016/j.cell.2014.11.021.
